# Supplementary figures and images for: Structures of G-protein coupled receptor HCAR1 in complex with Gi1 protein reveal the mechanistic basis for ligand recognition and agonist selectivity
Source: PLoS Biol. 2025 Apr 15;23(4):e3003126. doi: 10.1371/journal.pbio.3003126 (PMC12040280; doi:10.1371/journal.pbio.3003126)

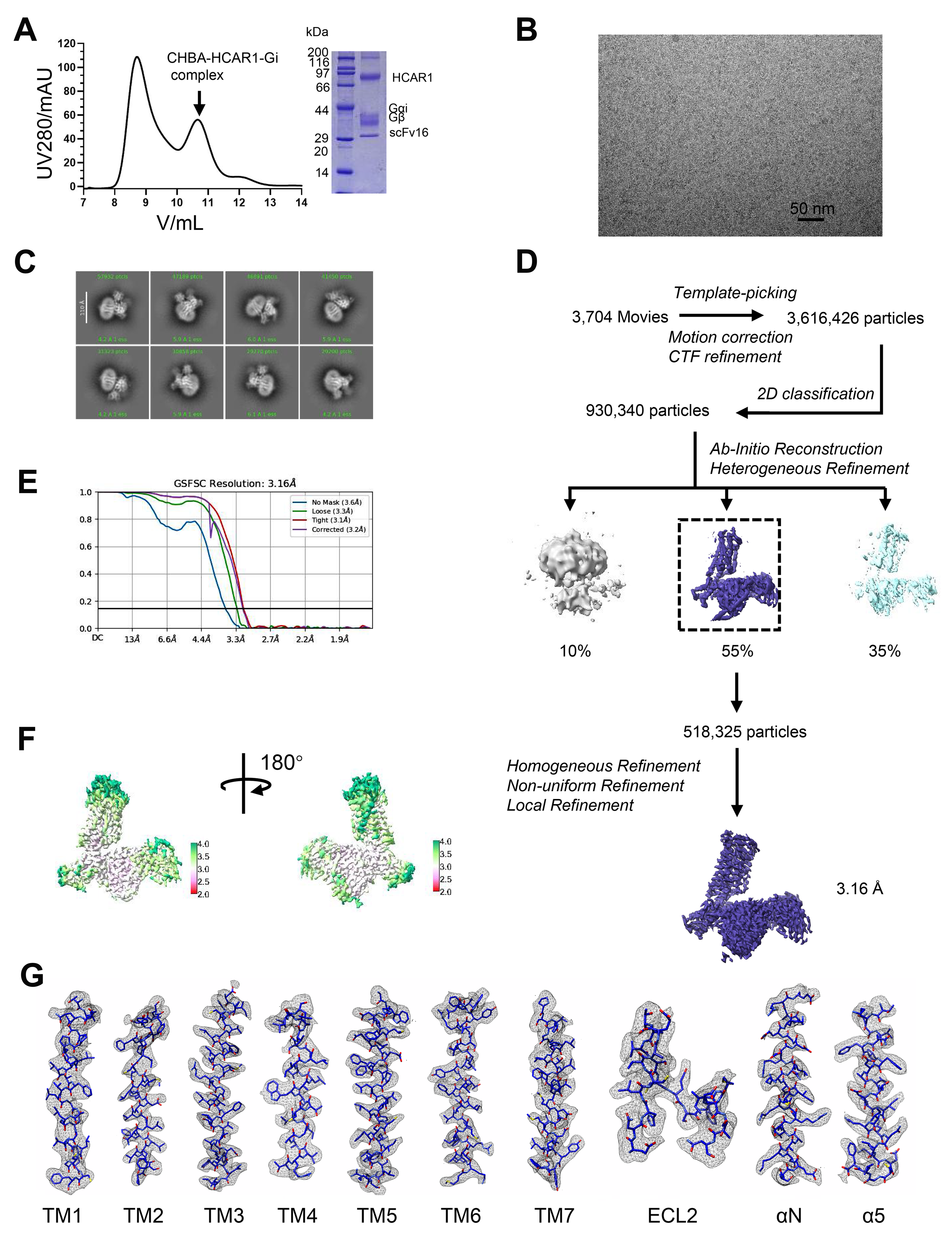

Supplement: S1 Fig — (A). Size exclusion chromatography profile and SDS–PAGE of the HCAR1-Gi1 complex. (B). Representative micrograph of the complex particles. (C). Representative 2D averages. (D). Workflow for cryo-EM image processing. (E). Gold-standard FSC curves of the 3D reconstructions. (F). Local resolution map of the complex. (G). Representative density maps and models for TM1–7 and ECL2 of HCAR1 and the α helices of Gαi1 (αN and α5). The original gel image can be found in S1 Raw Images. (TIF) [file pbio.3003126.s001.tif]

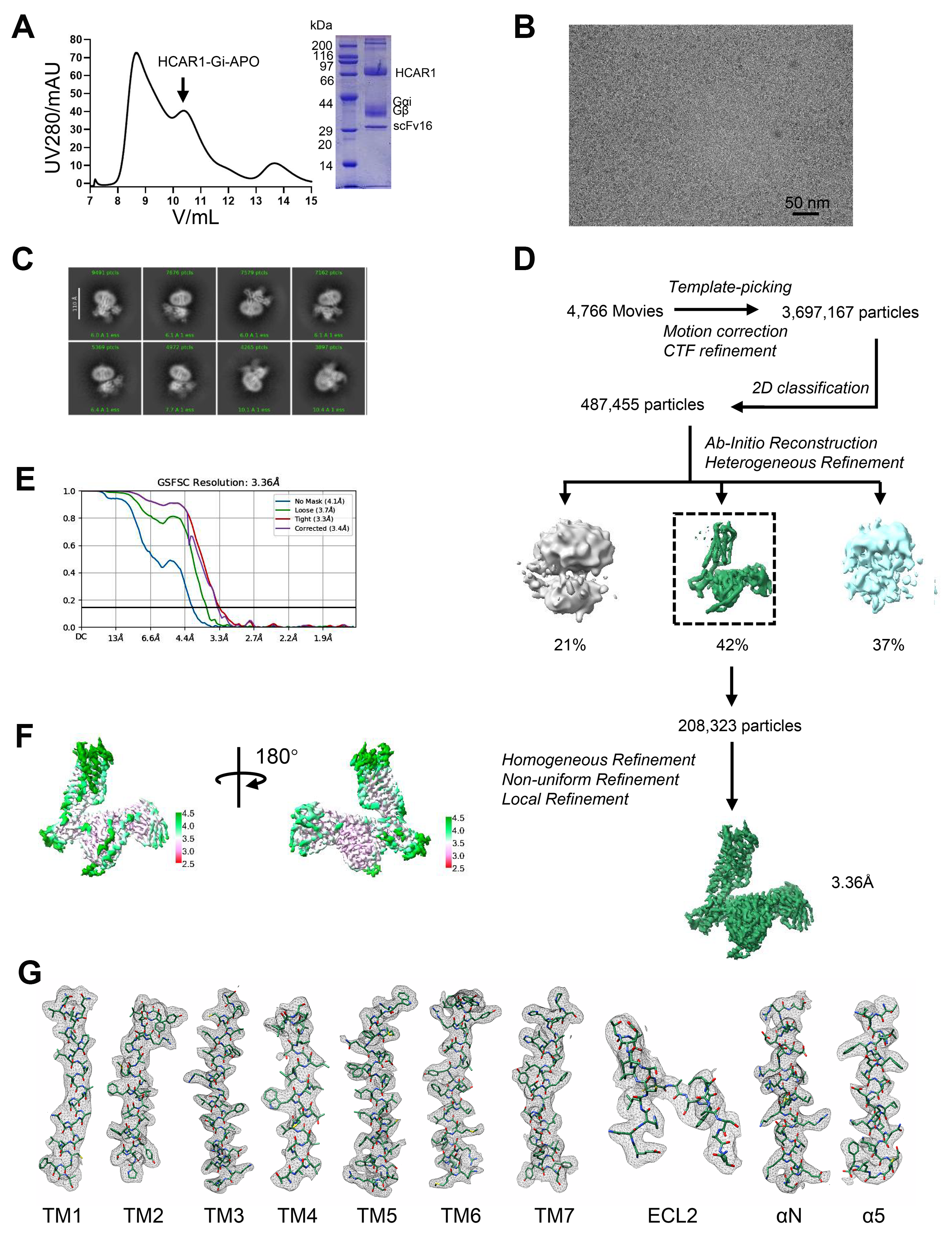

Supplement: S2 Fig — (A). Size exclusion chromatography profile and SDS–PAGE of the HCAR1-Gi1 complex. (B). Representative micrograph of the complex particles. (C). Representative 2D averages. (D). Workflow for cryo-EM image processing. (E). Gold-standard FSC curves of the 3D reconstructions. (F). Local resolution map of the complex. (G). Representative density maps and models for TM1–7 and ECL2 of HCAR1 and the α helices of Gαi1 (αN and α5). The original gel image can be found in S1 Raw Images. (TIF) [file pbio.3003126.s002.tif]

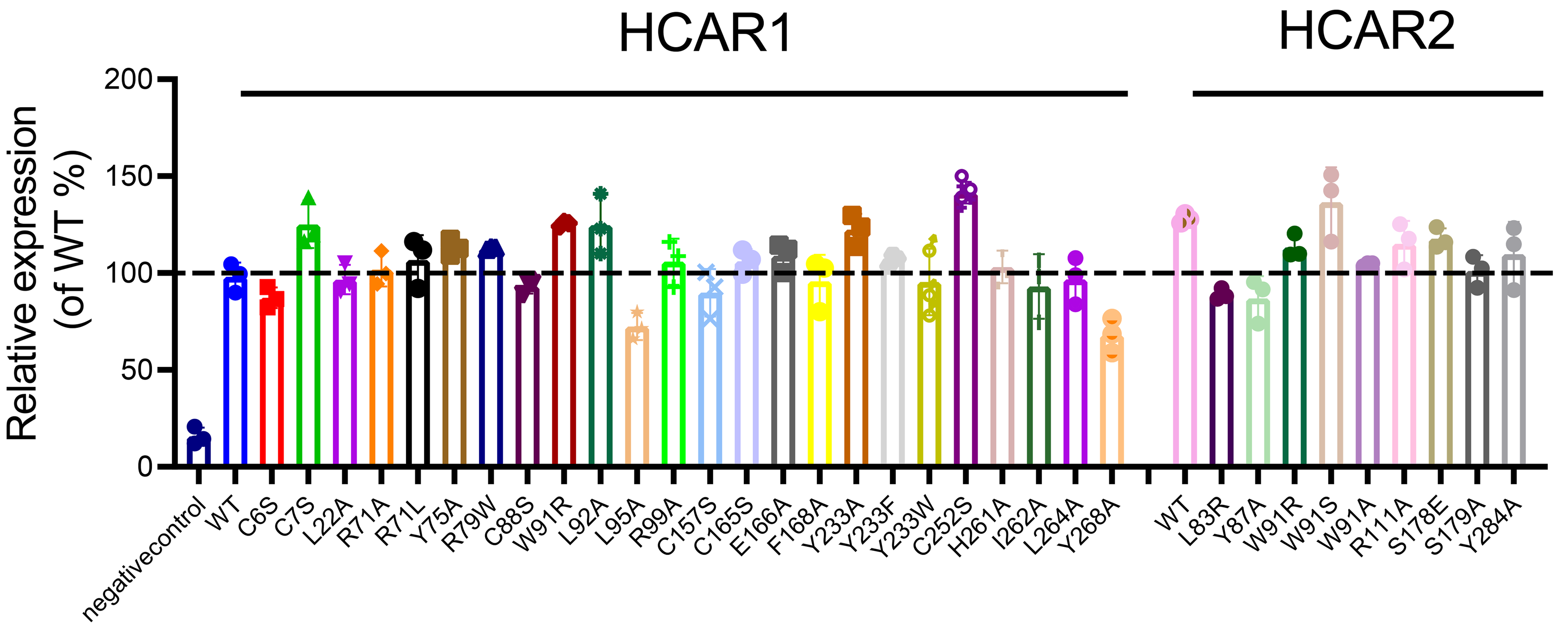

Supplement: S3 Fig — Relative cellular expression is determined by FACS analysis. The data are presented as means ± SEM. The experiments are performed in triplicate. The underlying data can be found in S1 Data. (TIF) [file pbio.3003126.s003.tif]

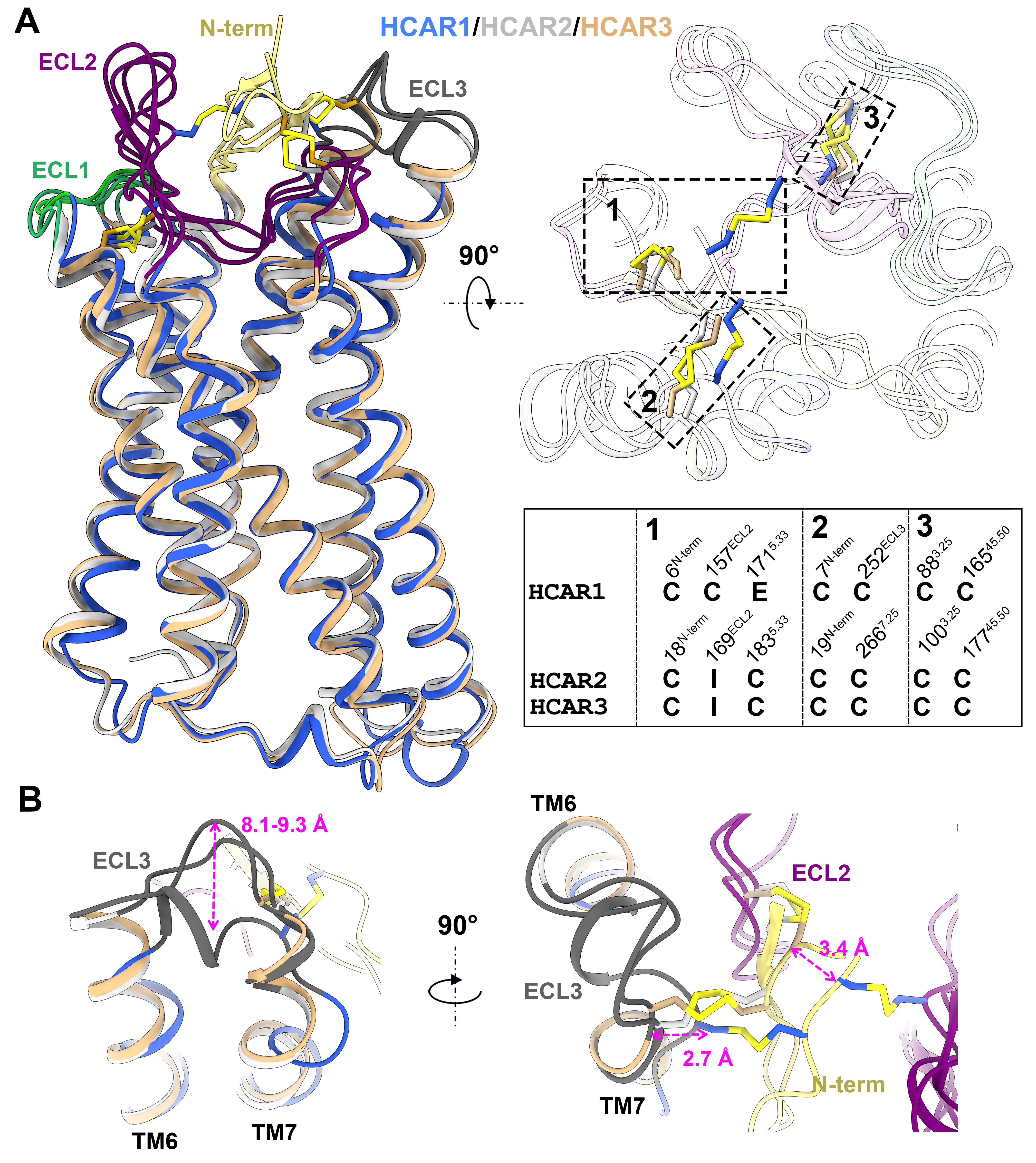

Supplement: S4 Fig — (A). Superposition of the extracellular architecture of HCAR1, HCAR2, and HCAR3. All HCAR receptors have three disulfide bonds and are divided into three groups: (1) CysN-term-CysECL2, (2) Cys45.50–Cys3.25, and (3) CysN-term–CysECL3. Groups 2 and 3 are conserved, while group 1 displays a difference in its spatial position. (B). Movement of the N-terminus and ECL3 in HCAR1 relative to those in HCAR2 and HCAR3. The structures of HCAR receptors are colored differently. Royal blue, HCAR1, light gray, HCAR2; sandy brown, HCAR3; khaki, N-terminal loop; pale green, ECL1; purple, ECL2; dark gray, ECL3; magenta arrow, shift in HCAR1 with respect to HCAR2 and HCAR3. (TIF) [file pbio.3003126.s004.tif]

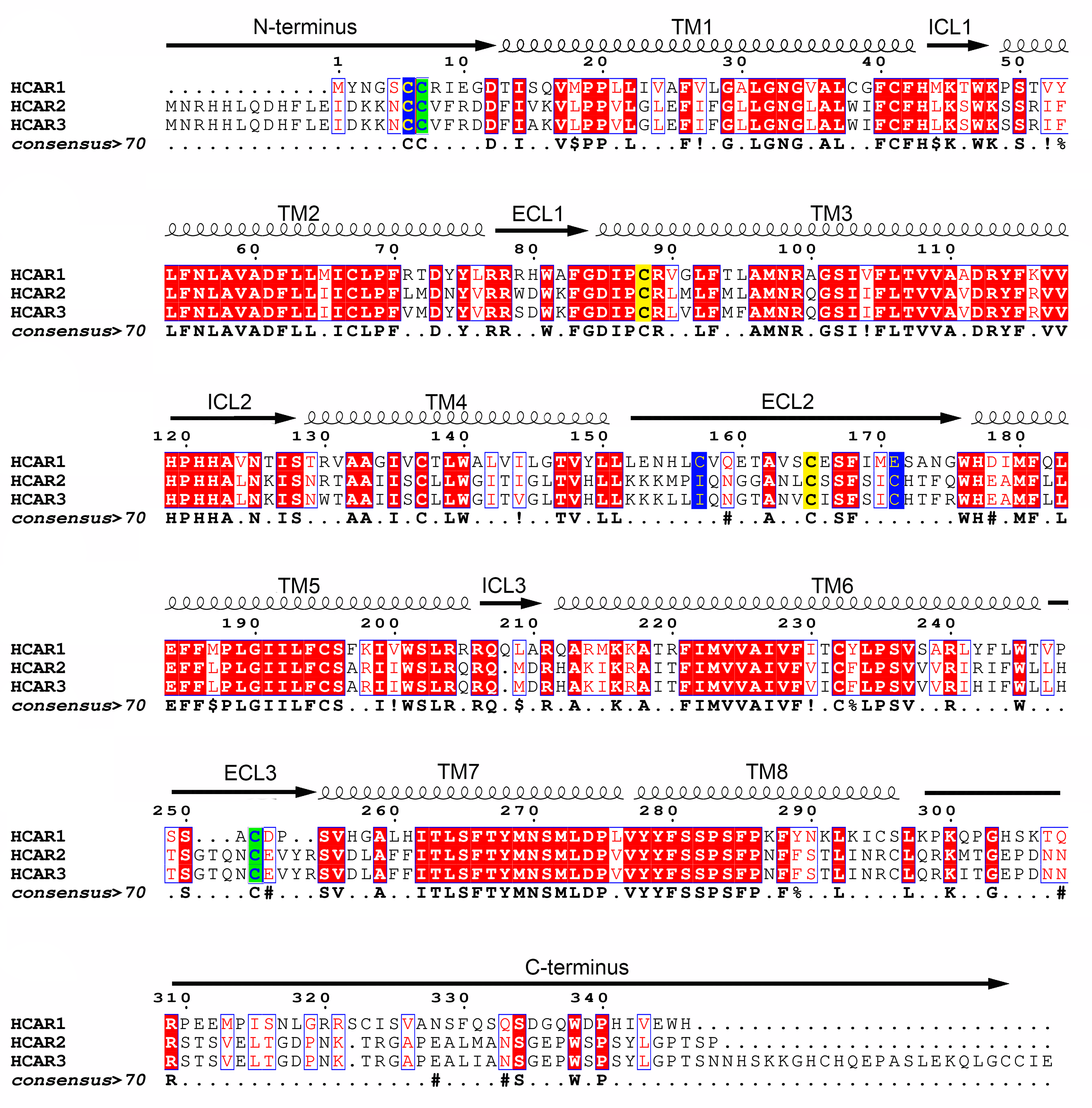

Supplement: S5 Fig — Positions that are identical between the receptors are highlighted with a red background. (TIF) [file pbio.3003126.s005.tif]

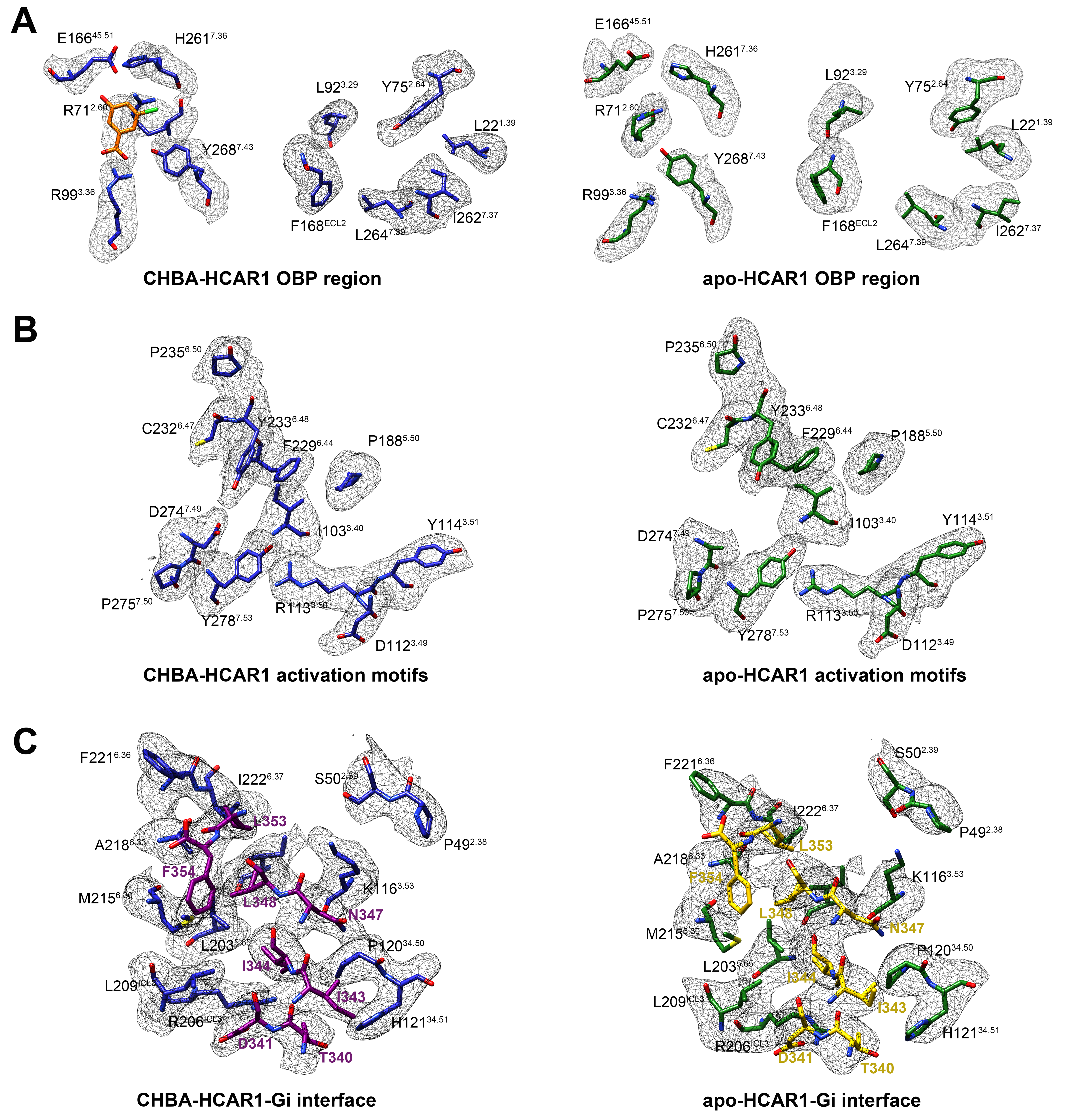

Supplement: S6 Fig — (A). Density maps of hydrophilic and hydrophobic residues in the OBP region. (B). Density maps of key activation motifs. (C). Density maps of key residues in the HCAR1-Gi1 interface. Royal blue-dark magenta, CHBA-HCAR1-Gi1; forest green-beige, apo-HCAR1-Gi1; orange, CHBA. (TIF) [file pbio.3003126.s006.tif]

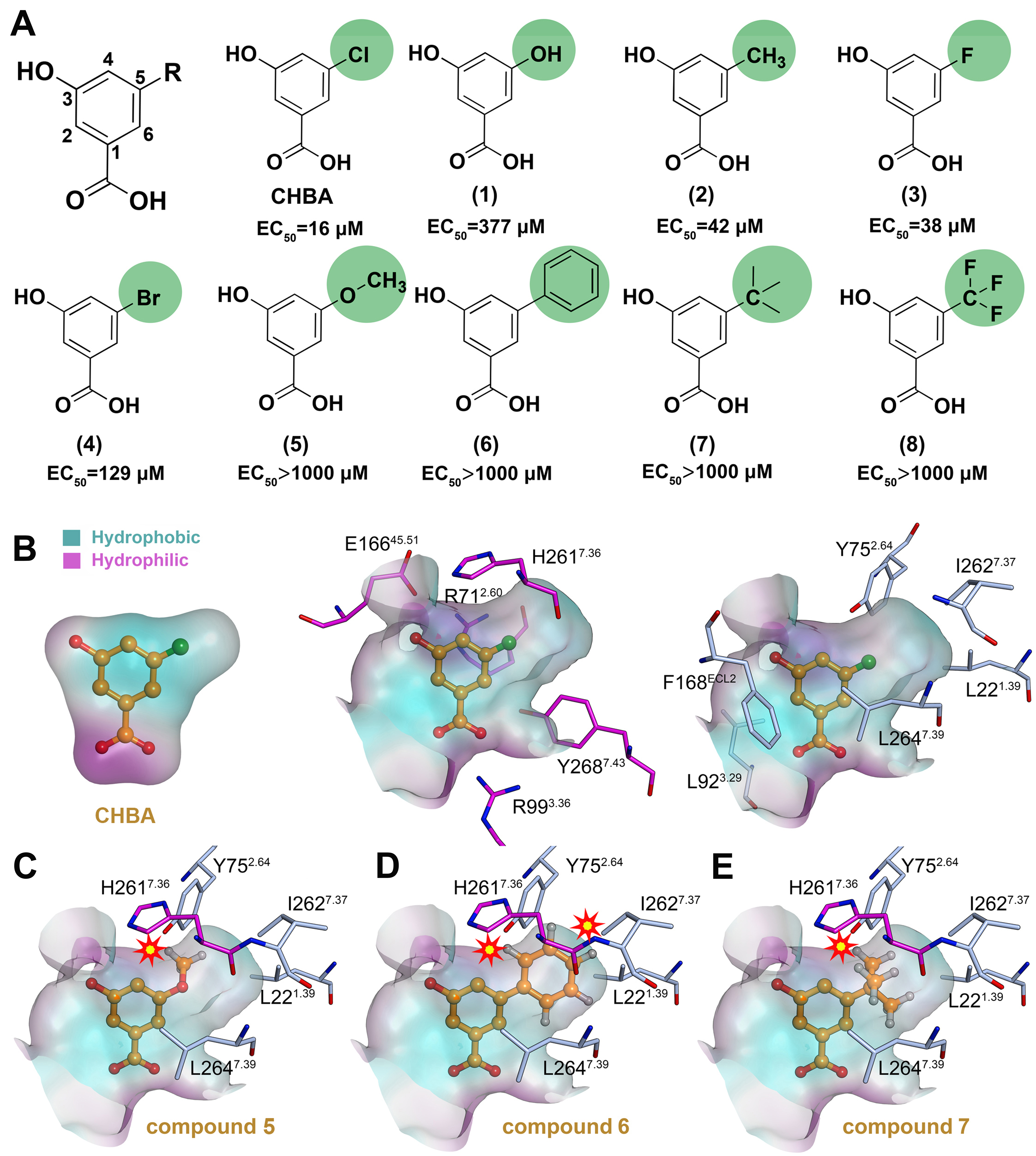

Supplement: S7 Fig — (A). Chemical structures of compounds substituted at CHBA’s 5-position. The EC50 values are obtained from the previous report [38]. (B). Hydrophilic and hydrophobic properties of CHBA and OBP in HCAR1. (C−E). Predicted binding modes of compound 5 (C), 6 (D), and 7 (E) with HCAR1. The pockets and residues are colored according to hydrophobicity (turquoise) and hydrophilicity (orchid). The red and yellow polygon represents the possible region of steric hindrance. (TIF) [file pbio.3003126.s007.tif]

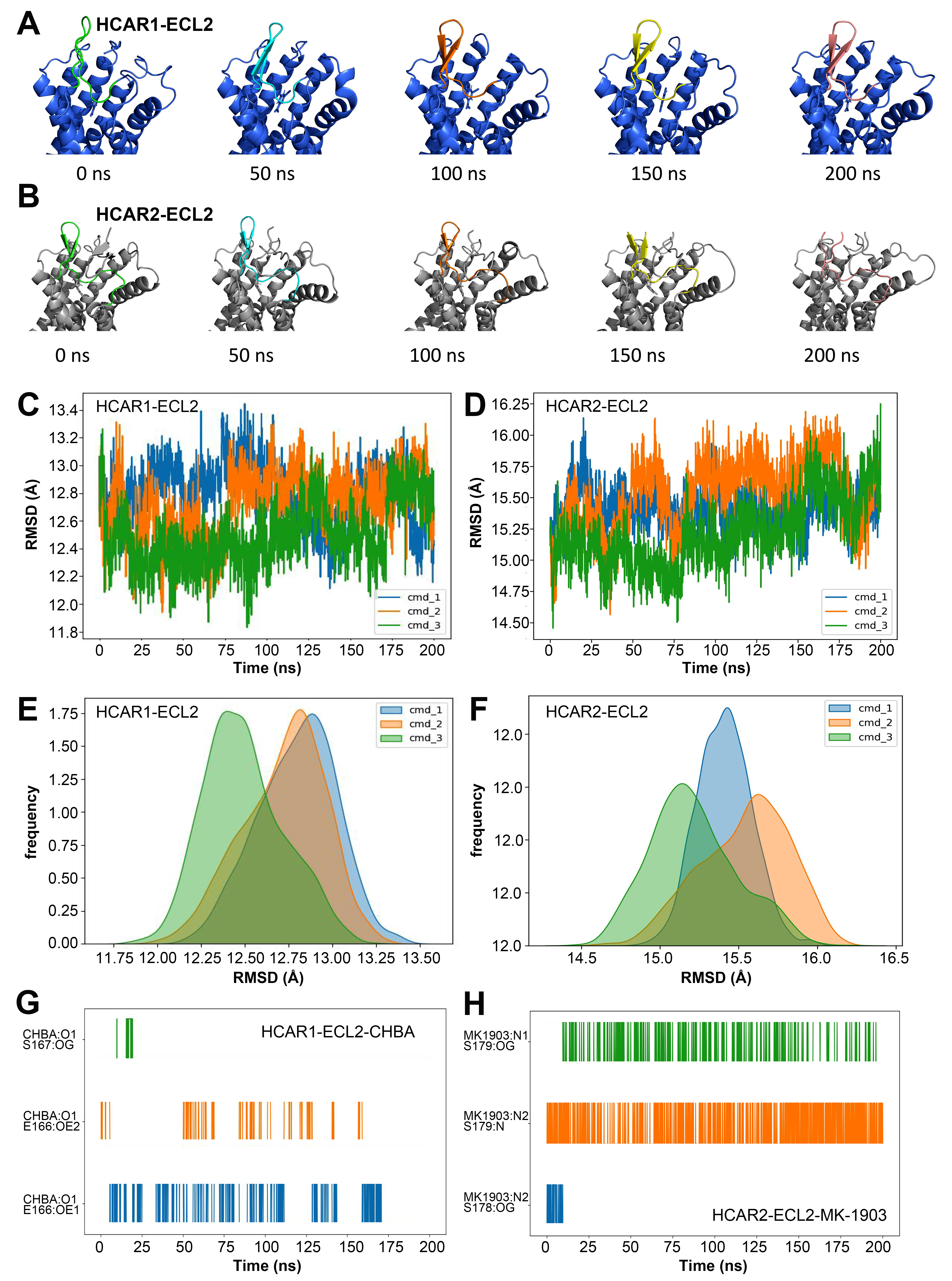

Supplement: S8 Fig — Conformational changes of ECL2 in the simulation trajectories of the HCAR1 (A) and HCAR2 (B) complexes at 0, 50, 100, 150, and 200 ns. RMSD of the ECL2 backbone atoms in the HCAR1 (C, E) and HCAR2 (D, F) complexes. The hydrogen bonds between ECL2 and ligand during the trajectories in HCAR1 (G) and HCAR2 (H) complexes. Royal blue, HCAR1; light gray, HCAR2. The underlying data can be found in S1 Data. (TIF) [file pbio.3003126.s008.tif]

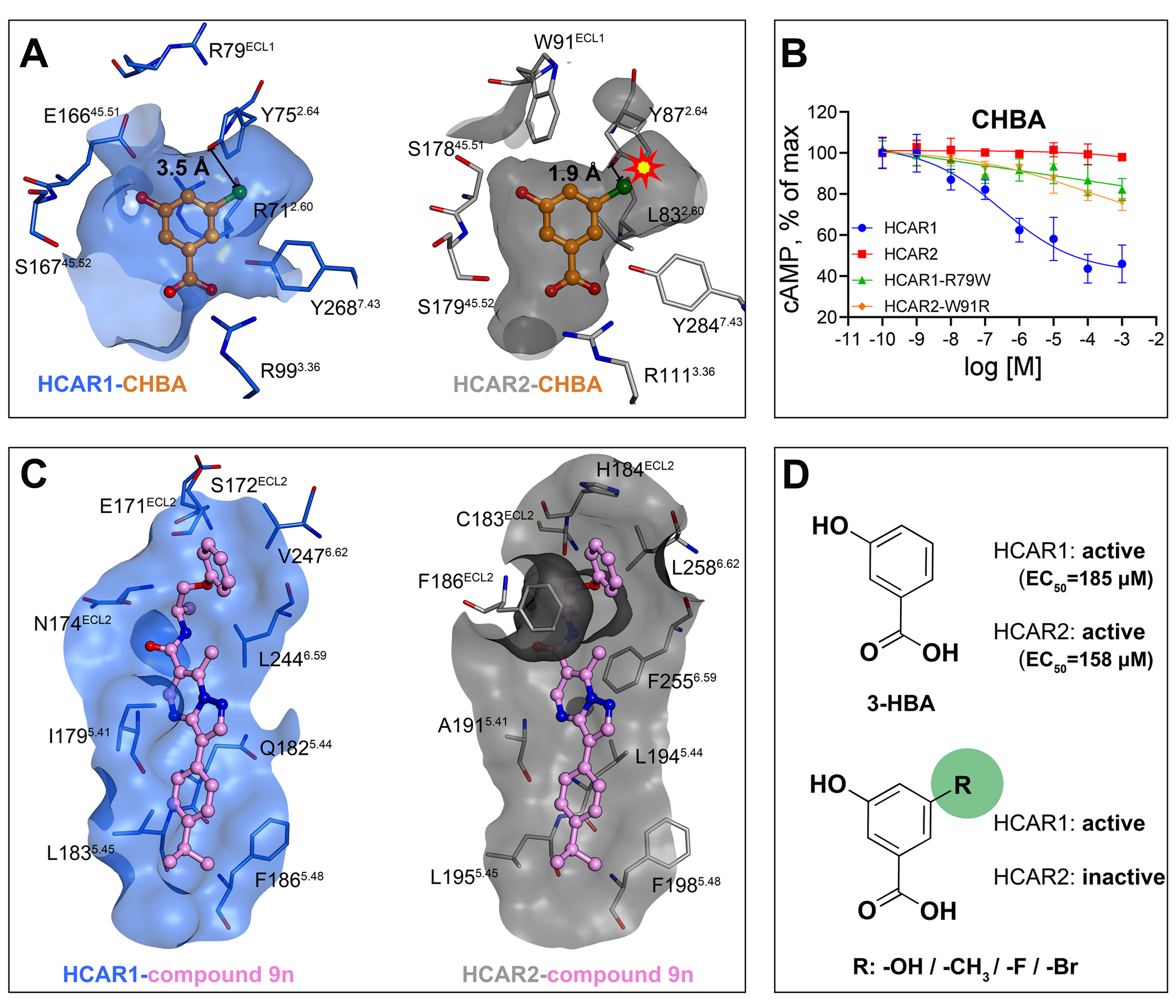

Supplement: S9 Fig — (A). Binding mode comparison of CHBA in OBP. (B). Effects on Gi-mediated cAMP by single point mutations of R79ECL1W in HCAR1 and W91ECL1R in HCAR2. The data are presented as means ± SEM. The experiments are performed in triplicate. The underlying data can be found in S1 Data. (C). Binding mode comparison of compound 9n in ABP. Royal blue, HCAR1; light gray, HCAR2; orange, CHBA; pink, compound 9n. (D) Selectivity of many other HCAR1 agonists. The EC50 values are obtained from the previous report [42]. (TIF) [file pbio.3003126.s009.tif]

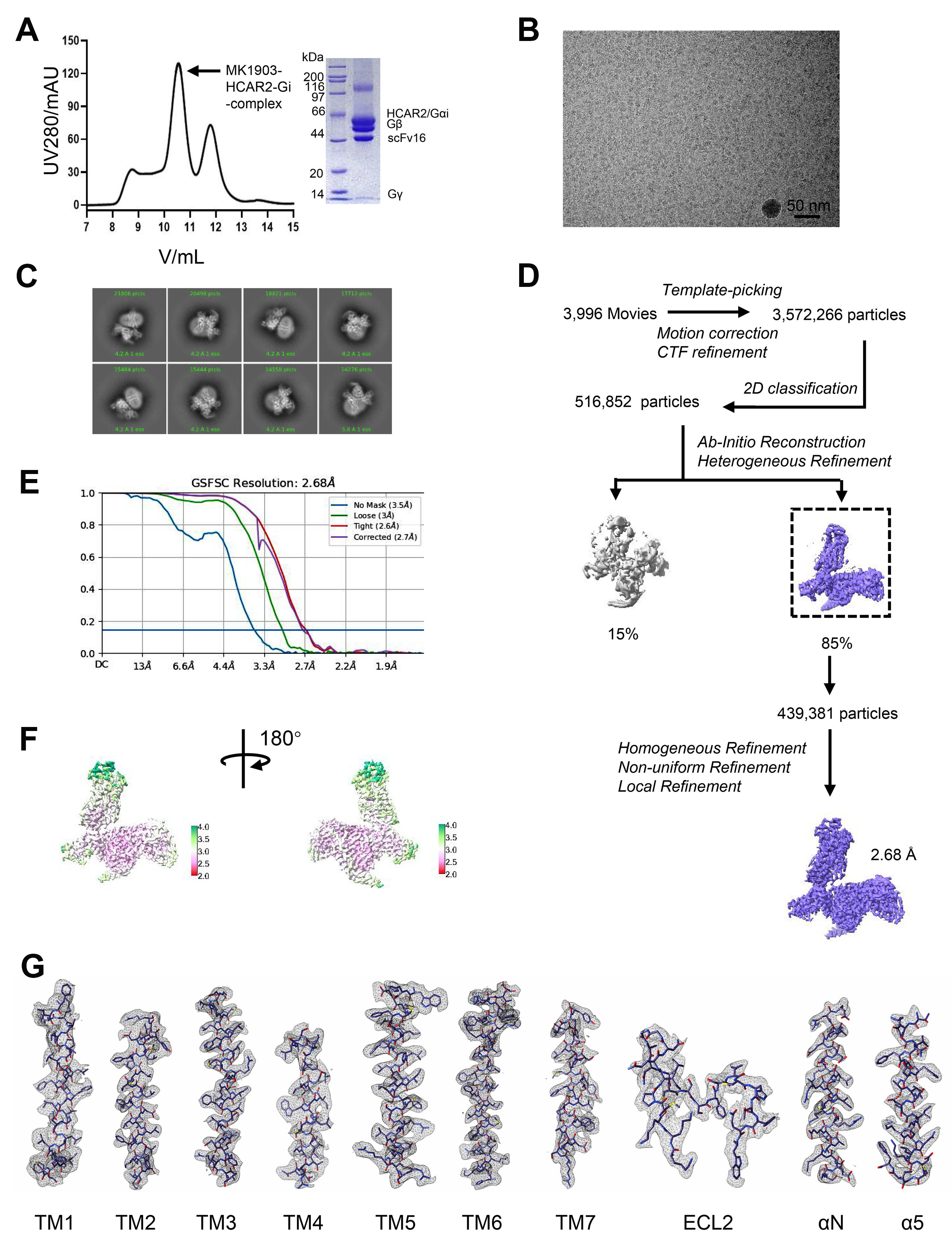

Supplement: S10 Fig — (A). Size exclusion chromatography profile and SDS–PAGE of the HCAR2-Gi1 complex. (B). Representative micrograph of the complex particles. (C). Representative 2D averages. (D). Workflow for cryo-EM image processing. (E). Gold-standard FSC curves of the 3D reconstructions. (F). Local resolution map of the complex. (G). Representative density maps and models for TM1–7 and ECL2 of HCAR2 and the α helices of Gαi1 (αN and α5). The original gel image can be found in S1 Raw Images. (TIF) [file pbio.3003126.s010.tif]

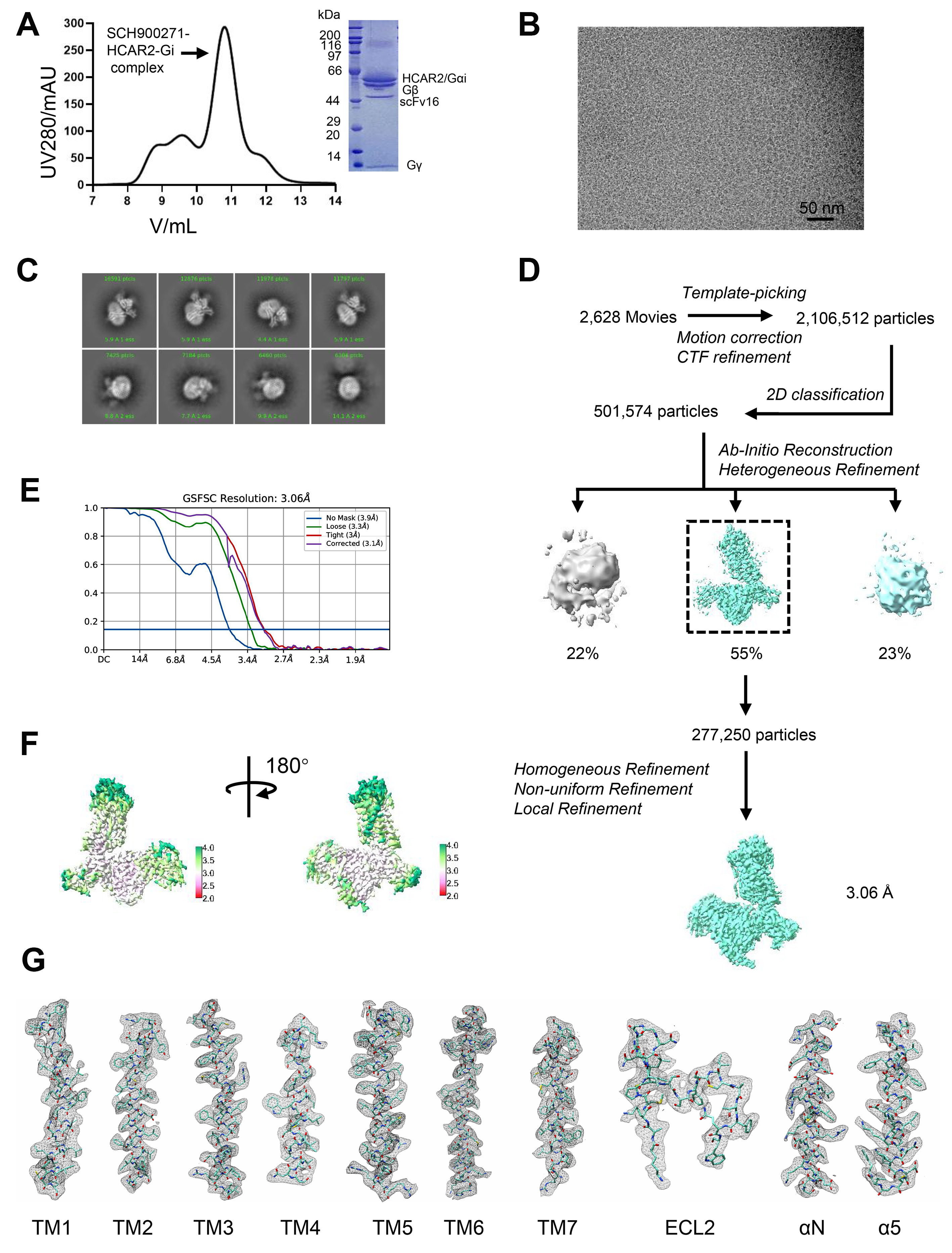

Supplement: S11 Fig — (A). Size exclusion chromatography profile and SDS–PAGE of the HCAR2-Gi1 complex. (B). Representative micrograph of the complex particles. (C). Representative 2D averages. (D). Workflow for cryo-EM image processing. (E). Gold-standard FSC curves of the 3D reconstructions. (F). Local resolution map of the complex. (G). Representative density maps and models for TM1–7 and ECL2 of HCAR2 and the α helices of Gαi1 (αN and α5). The original gel image can be found in S1 Raw Images. (TIF) [file pbio.3003126.s011.tif]

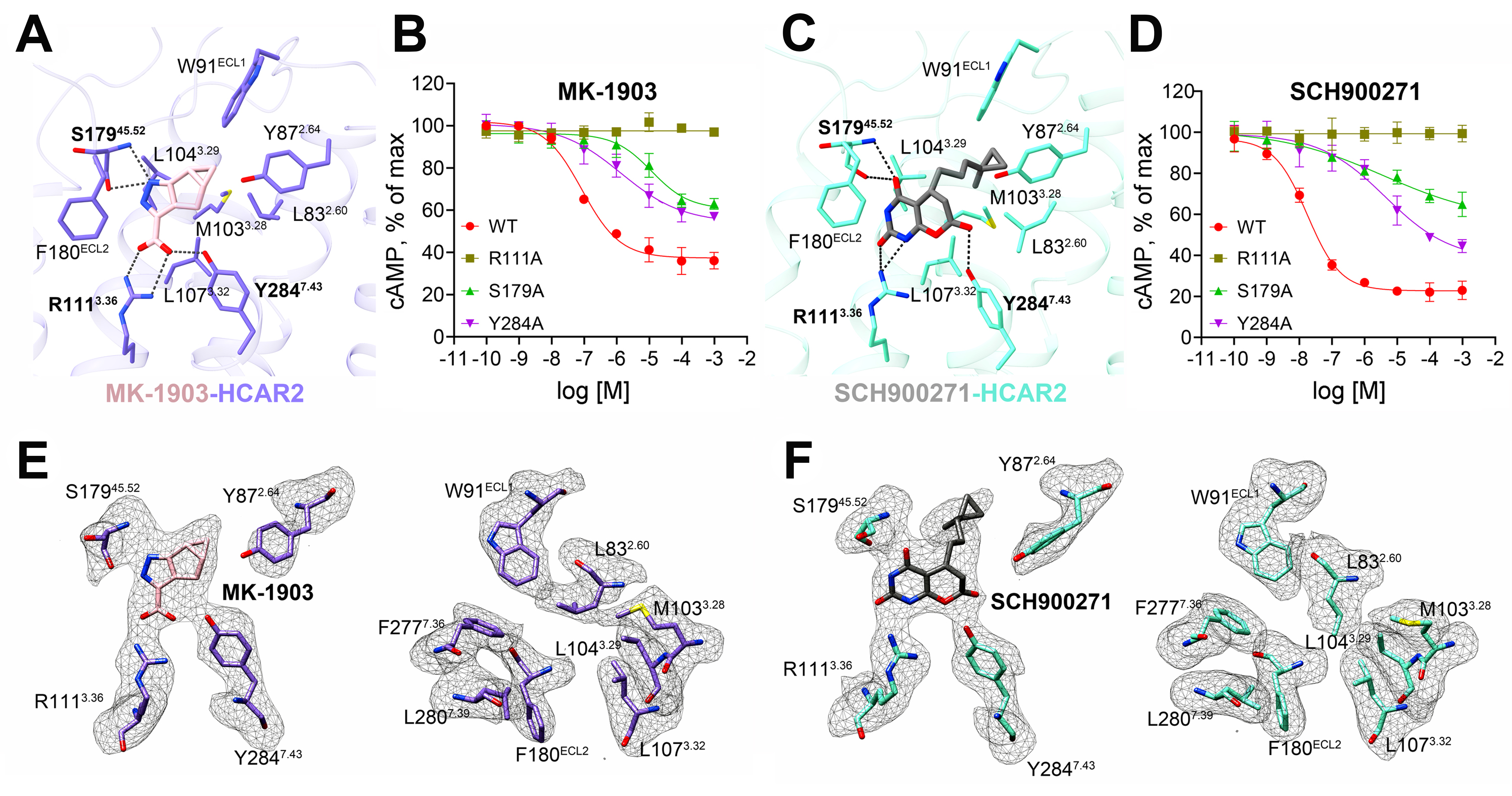

Supplement: S12 Fig — (A, B). Specific interactions of MK-1903 with HCAR2. Effects on Gi-mediated cAMP by single point mutations of key residues that interact with MK-1903. (C, D). Specific interactions of SCH900271 with HCAR2. Effects on Gi-mediated cAMP by single point mutations of key residues that interact with SCH900271. The data are presented as means ± SEM. The experiments are performed in triplicate. The underlying data can be found in S1 Data. (E, F) Density maps of MK-1903, SCH900271, and surrounding key residues. The ligands and residues are shown using a stick representation. Pink-medium slate blue, MK-1903-HCAR2; gray-aquamarine, SCH900271-HCAR2. (TIF) [file pbio.3003126.s012.tif]

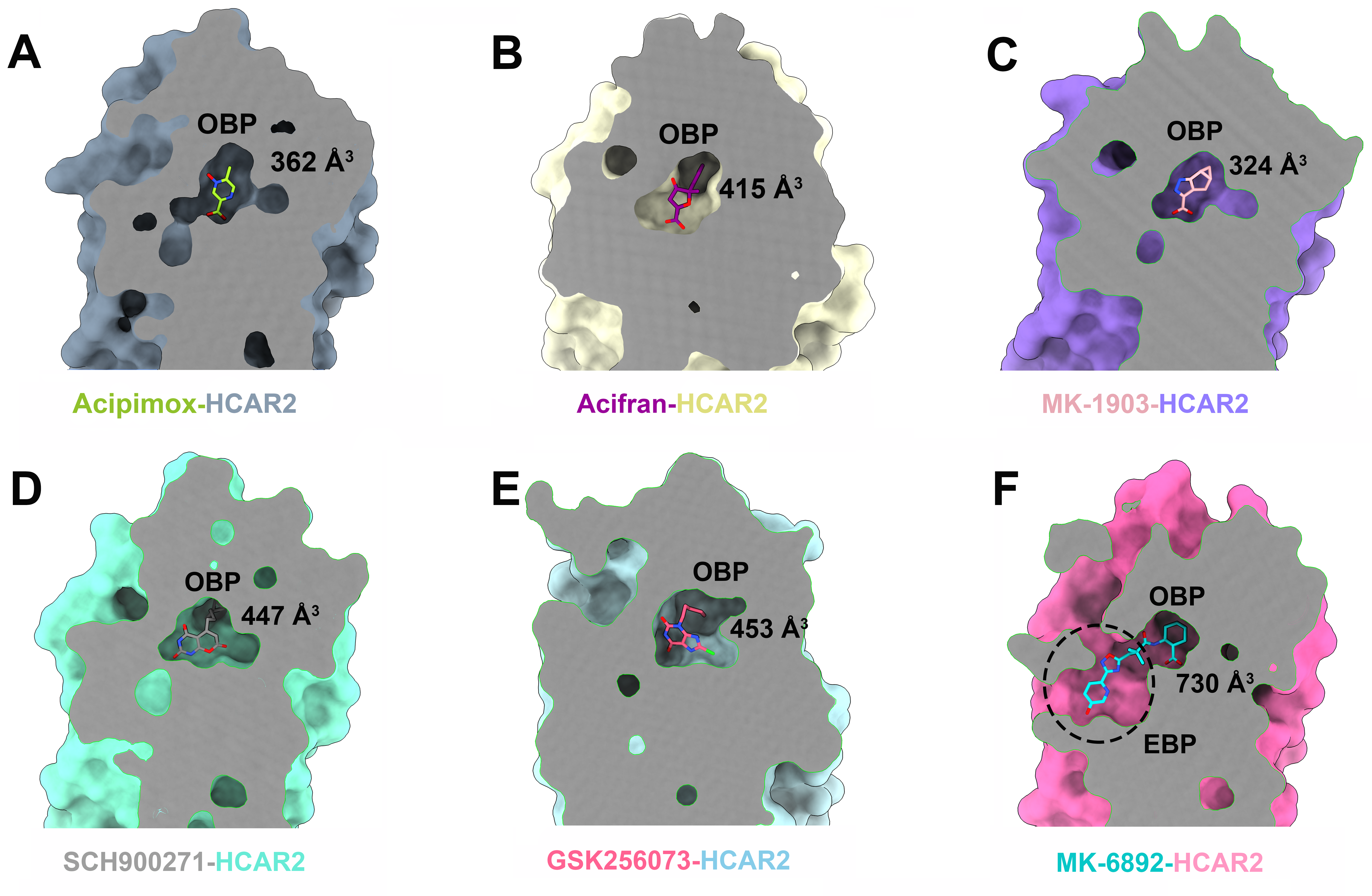

Supplement: S13 Fig — (A−F). Different binding modes of HCAR2 with acipimox (PDB: 8I7V) (A), acifran (PDB: 8IHI) (B), MK-1903 (C), SCH900271 (D), GSK256073 (PDB: 8I7W) (E), and MK-6892 (PDB: 8IJD) (F). Except for MK-6892, all other agonists bind exclusively to the OBP region. Yellow green-slate gray, acipimox-HCAR2; purple-lemon chiffon, acifran-HCAR2; pink-medium slate blue, MK-1903-HCAR2; gray-aquamarine, SCH900271-HCAR2; rose red-light sky blue, GSK256073-HCAR2; cyan-light magenta, MK-6892-HCAR2. (TIF) [file pbio.3003126.s013.tif]

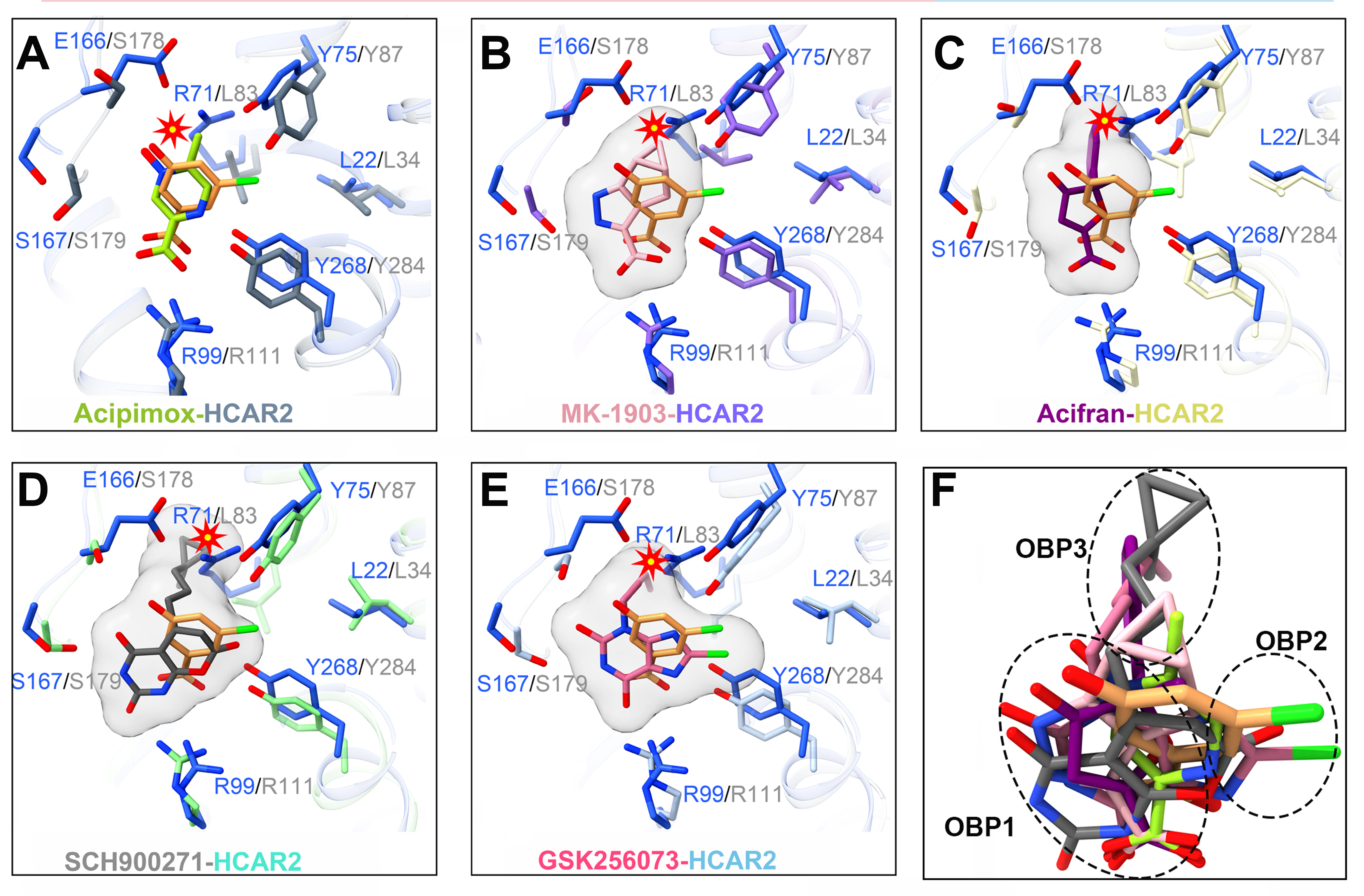

Supplement: S14 Fig — (A−E). Pairwise comparisons of the CHBA-HCAR1 versus acipimox-HCAR2 (A), MK-1903-HCAR2 (B), acifran-HCAR2 (C), SCH900271-HCAR2 (D), and GSK256073-HCAR2 (E). Orange-royal blue, CHBA-HCAR1; yellow green-slate gray, acipimox-HCAR2; pink-medium slate blue, MK-1903-HCAR2; purple-lemon chiffon, acifran-HCAR2; gray-aquamarine, SCH900271-HCAR2; rose red-light sky blue, GSK256073-HCAR2. (F). Superposition of the subtype-specific agonists of HCAR1 and HCAR2. (TIF) [file pbio.3003126.s014.tif]

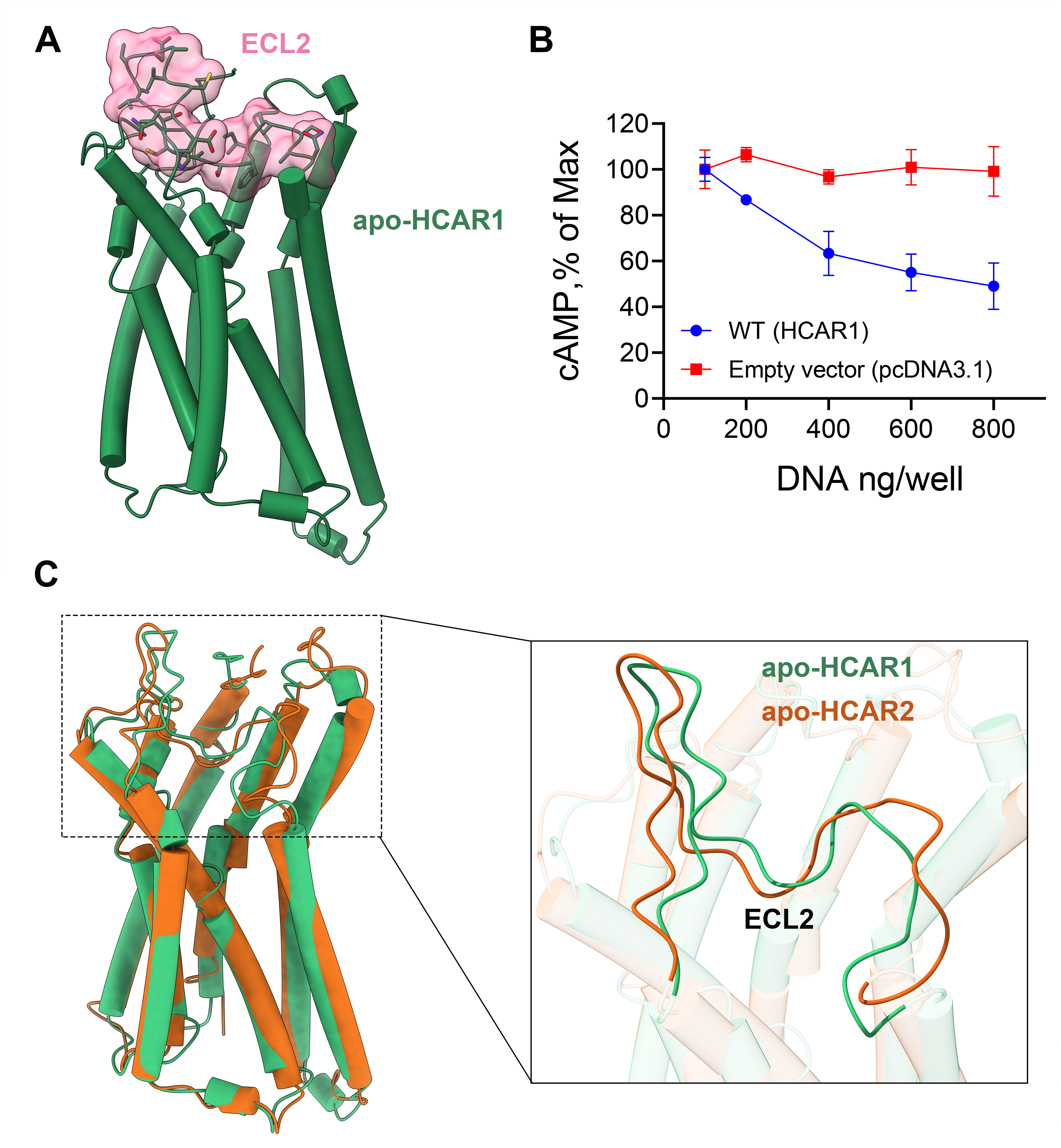

Supplement: S15 Fig — (A). ECL2 region in the apo state of HCAR1. (B). Effects of increasing HCAR1 receptor concentration on Gi-mediated cAMP. The data are presented as means ± SEM. The experiments are performed in triplicate. The underlying data can be found in S1 Data. (C). Superposition of the ECL2 regions in the apo states of HCAR1 and HCAR2. Forest green, apo-HCAR1; chocolate, apo-HCAR2. (TIF) [file pbio.3003126.s015.tif]

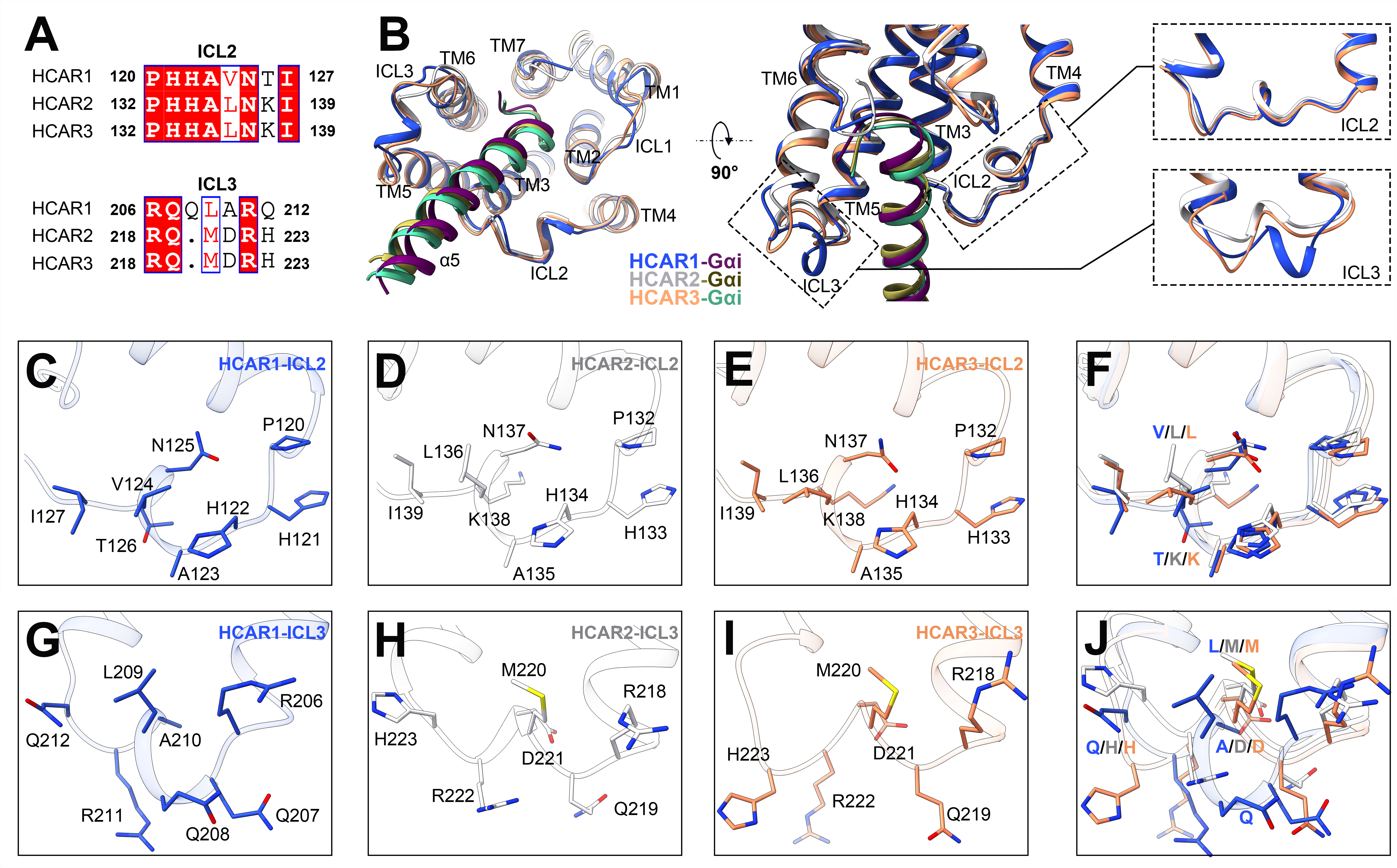

Supplement: S16 Fig — (A). Sequence alignment of ICL2 and ICL3 regions in HCAR1–3. Positions that are identical between the receptors are highlighted with a red background. (B). Superposition of the architecture of ICL2 and ICL3 regions in HCAR1–3. (C−F). Detailed distribution of amino acids in the ICL2 of HCAR1–3. (G−J). Detailed distribution of amino acids in the ICL3 of HCAR1–3. Royal blue-dark magenta, CHBA-HCAR1-Gi1; light gray-dark khaki, HCAR2-Gi1; sandy brown-medium aquamarine, HCAR3-Gi1. (TIF) [file pbio.3003126.s016.tif]

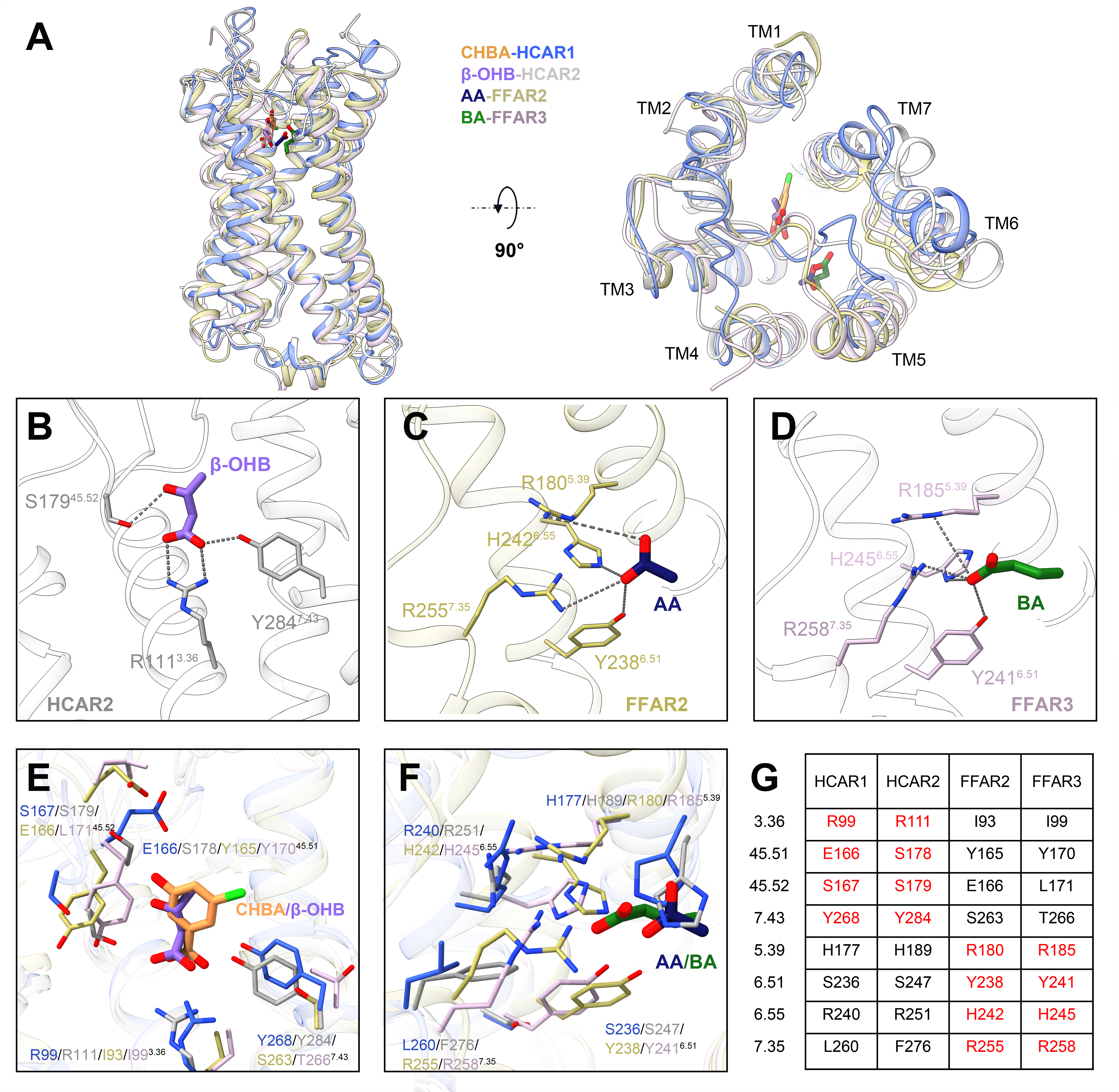

Supplement: S17 Fig — (A). Superposition of the architecture of CHBA-HCAR1, β-OHB-HCAR2, AA-FFAR2, and BA-FFAR3. (B). Detailed polar interactions of β-OHB with HCAR2. (C). Detailed polar interactions of AA with FFAR2. (D). Detailed polar interactions of BA with FFAR3. (E). Comparison of the key amino acids responsible for ligand recognition in the OBP region of HCAR1 and HCAR2. (F). Comparison of the key amino acids responsible for ligand recognition in the OBP region of FFAR2 and FFAR3. (G). Sequence alignment of key amino acids in HCAR1/2 and FFAR2/3. Orange-royal blue, CHBA-HCAR1; medium purple-light gray, β-OHB-HCAR2; dark blue-dark khaki, AA-FFAR2; dark green-thistle, BA-FFAR3. (TIF) [file pbio.3003126.s017.tif]

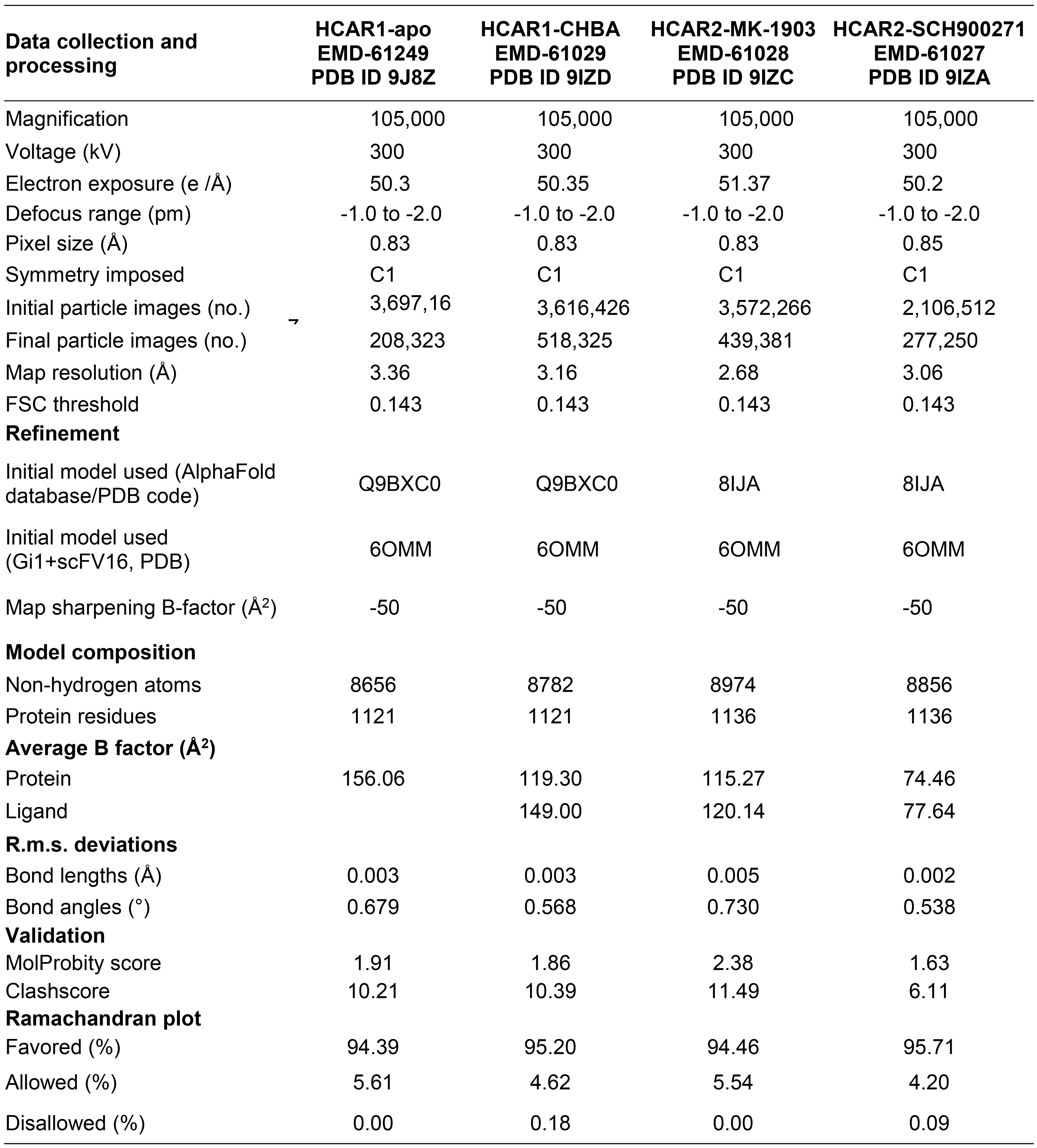

Supplement: S1 Table — (TIF) [file pbio.3003126.s018.tif]
