# Supplementary material for: Structures of G-protein coupled receptor HCAR1 in complex with Gi1 protein reveal the mechanistic basis for ligand recognition and agonist selectivity
Source: PLoS Biol. 2025 Apr 15;23(4):e3003126. doi: 10.1371/journal.pbio.3003126 (PMC12040280; doi:10.1371/journal.pbio.3003126)
Supplement: S1 Raw Images — (PDF) [file pbio.3003126.s019.pdf]

Fig. S1A

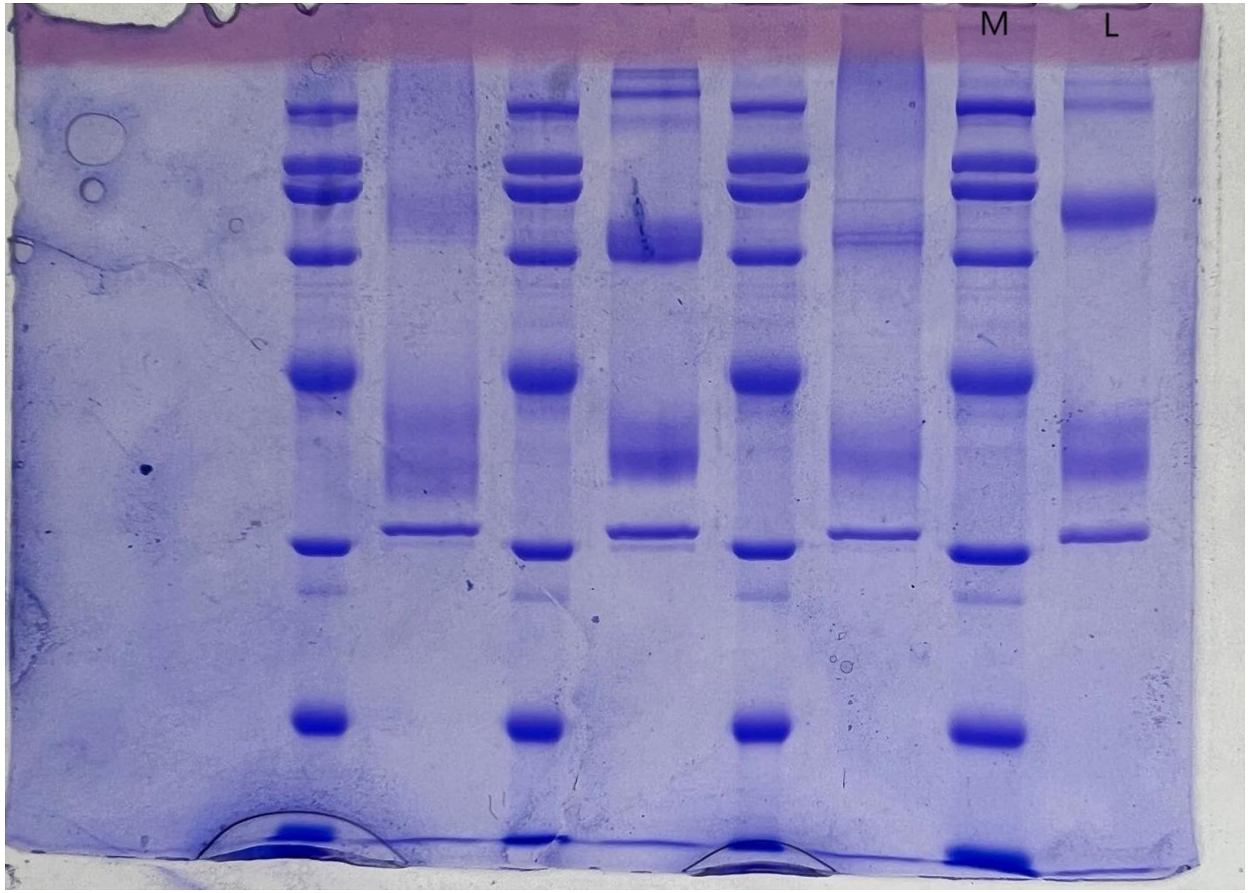

M: Marker (Protein Ladder)
L: Sample loaded

Fig. S2A

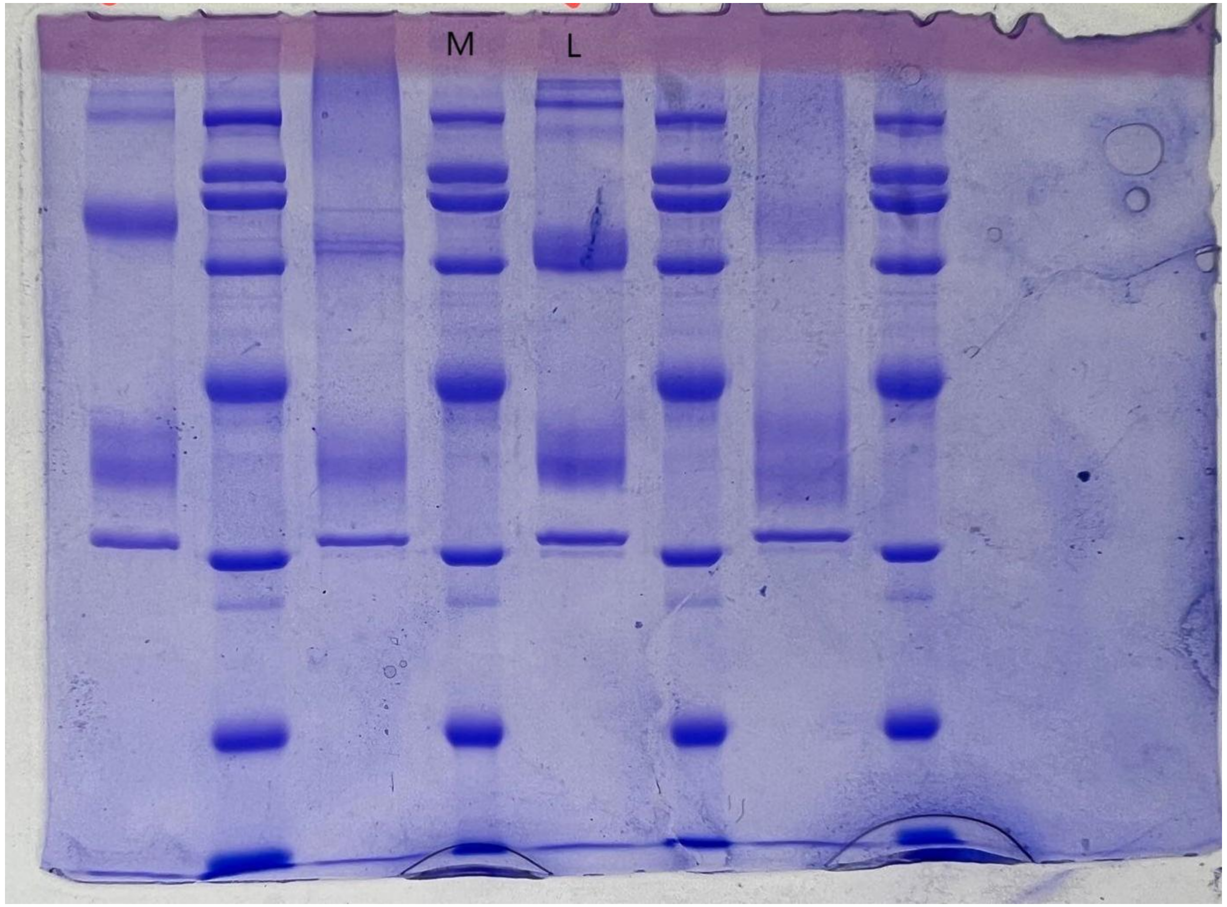

M: Marker (Protein Ladder)
L: Sample loaded

Fig. S10A

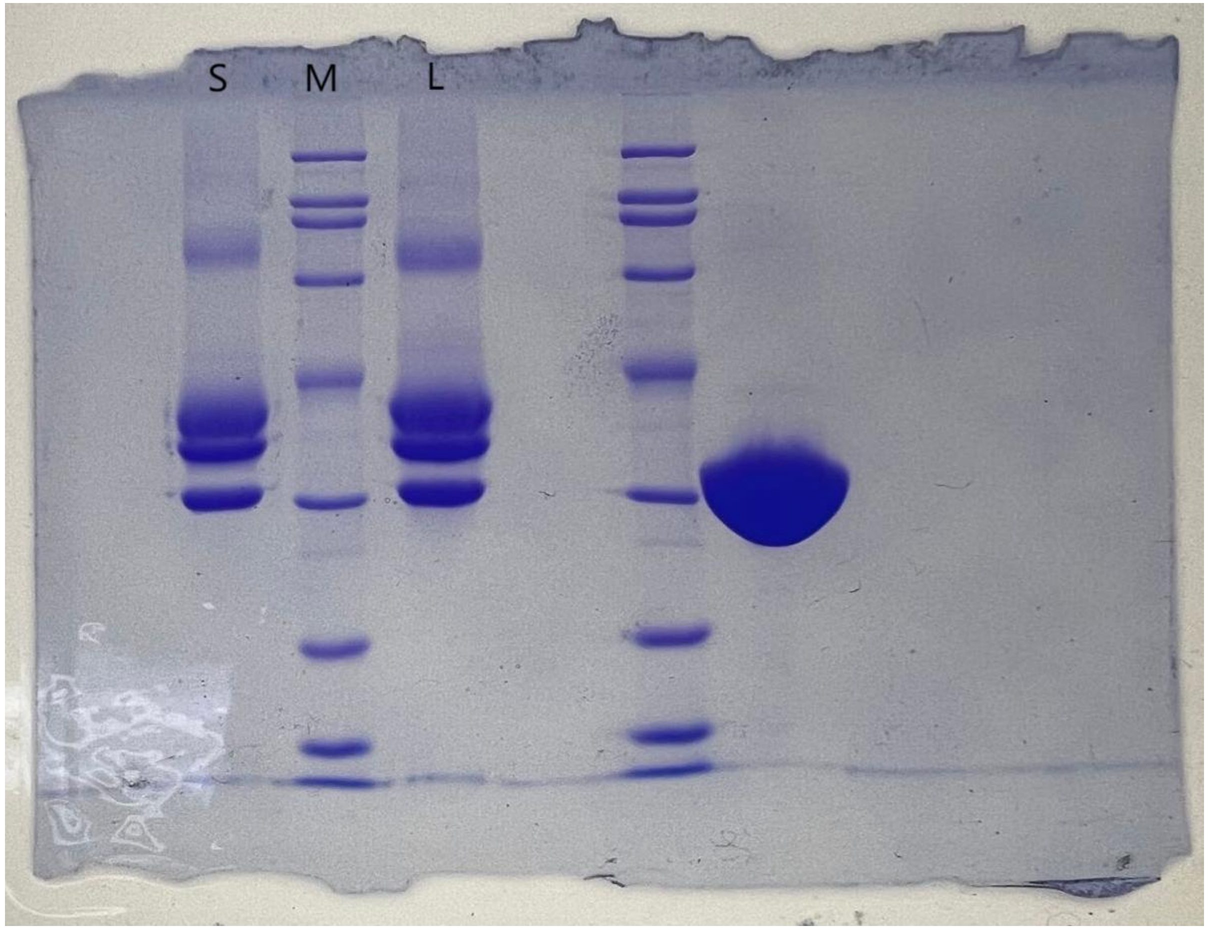

M: Marker (Protein Ladder)
L: Sample loaded
S: Sample collected with different concentration

Fig. S11A

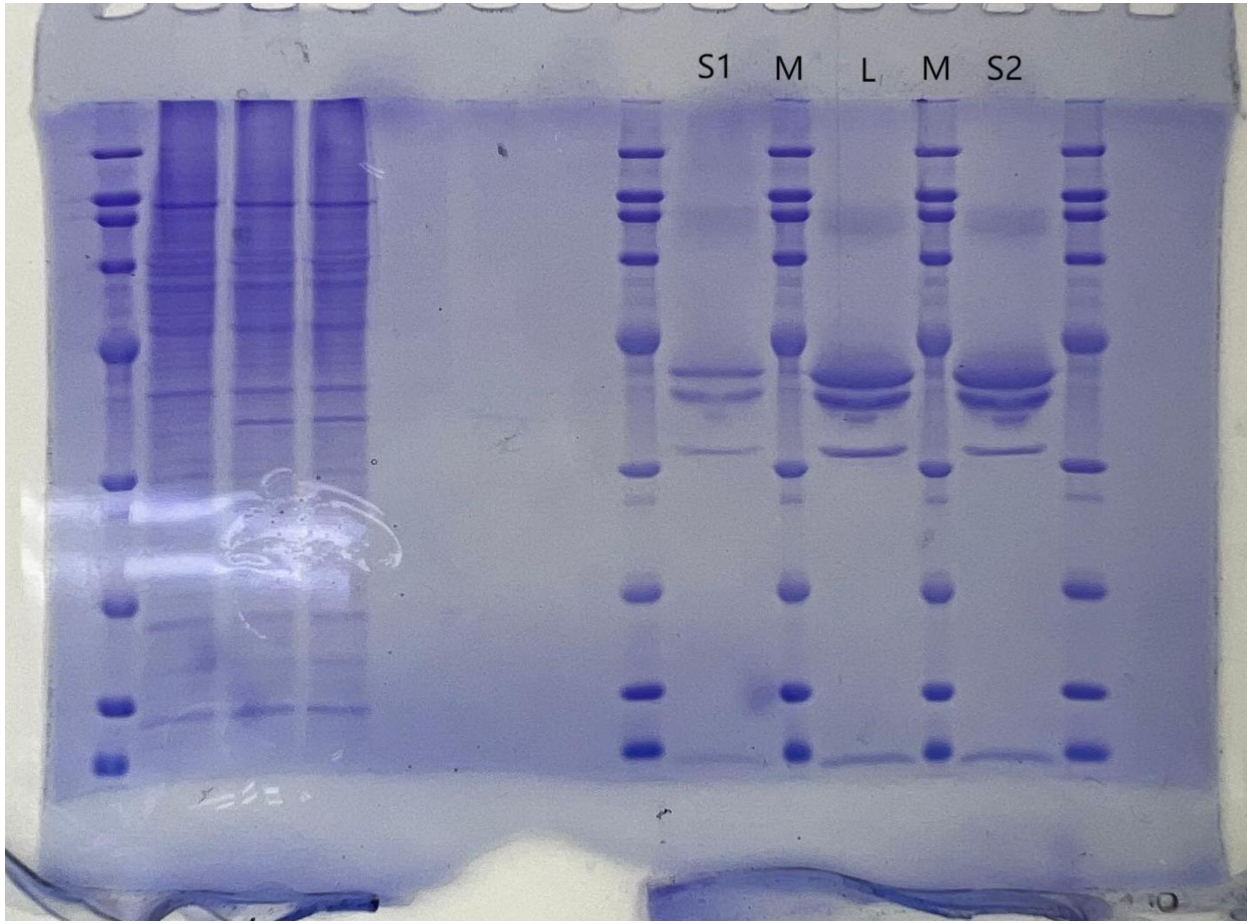

M: Marker (Protein Ladder)
L: Sample loaded
S1,S2: Sample collected with different concentration
