# Supplementary material for: Structures of G-protein coupled receptor HCAR1 in complex with Gi1 protein reveal the mechanistic basis for ligand recognition and agonist selectivity
Source: PLoS Biol. 2025 Apr 15;23(4):e3003126. doi: 10.1371/journal.pbio.3003126 (PMC12040280; doi:10.1371/journal.pbio.3003126)
Supplement: Attachment — Submitted filename: Response letter-PLOS Biology.pdf [file pbio.3003126.s023.pdf]

香港中文大學（深圳）
THE CHINESE UNIVERSITY OF HONG KONG, SHENZHEN

Dear Editors and Reviewers,

We would like to submit our revision for manuscript PBIOLOGY-D-24-02983R1 entitled "**Molecular basis for ligand recognition and selectivity of human lactate receptor HCAR1**". We really appreciate the time both you and the reviewers have taken to detail your helpful comments and suggestions. In this revised version, we have carefully modified our original manuscript according to your comments and suggestions, and have also referred to the regulations and format of your distinguished journal. Below you will find a list of changes for the point-by-point responses to the reviewers' comments and a list of answers to the questions on the checklist. The corrections were highlighted in yellow in the revised manuscript. We hope that the quality of our manuscript would meet the publication standard of ***PLOS Biology.***

Yang Du

Sincerely,

Yang Du, PhD, Assistant Professor/PI
School of Medicine
Kobilka Institute of Innovative Drug Discovery
The Chinese University of Hong Kong,Shenzhen 518172, China

List of Changes

(Manuscript ID: PBIOLOGY-D-24-02983R1, Pan et al.)

Response to Reviewer 1

Question 1. There are disordered amino acid residues in the structures of HCAR1-CHBA and HCAR2-SCH900271 in Supplementary Table S1, and the names of these residues should be clearly stated in the main text.

Answer 1. Thank you very much for your valuable suggestion. According to your requirement, we have added the disordered amino acid residues in the main text.

Notably, a few residues observed in the disallowed regions are from the structures of CHBA-bound HCAR1 (D12 and P69) and SCH900271-bound HCAR2 (L245). (Please see line 506-507)

Question 2. In 'Model building and refinement' you say that the structures of HCAR1 and 2 were analyzed using the predicted structure from AlphaFold as the initial model. There must be structures already analyzed.

Answer 2. Thank you very much for your kind and professional comments and suggestions. The models of MK-1903- and SCH900271-bound HCAR2 were built with the reported niacin-bound HCAR2 cryo-EM structure (PDB: 8IJA) as the template. However, no cryo-EM structures have yet been resolved for HCAR1. Therefore, the models of apo and CHBA-bound HCAR1 were constructed using the initial template from the AlphaFold Protein Structure Database (ID: Q9BXC0). According to your requirement, we have revised the related methods in the manuscript. (Please see line 497-500)

Response to Reviewer 2

Question 1. Some of the ligands, such as CHBA and MK1903, are relatively small. The cryo-EM density alone does not allow for unambiguous modeling based solely on the density map. How did the authors ensure that the orientations of these ligands are correct? Is it possible for both ligands to be positioned in the opposite direction?

Answer 1. Thank you very much for your kind and professional comments and suggestions. The ligands, such as CHBA and MK1903, are indeed small. Despite this, according to the density map, the orientation of MK-1903 can be determined. When MK-1903 adopts the reverse direction, the ligand cannot fully match the density (Fig A and B). However, the density map for CHBA is a bit ambiguous. It seems that both the forward and reverse directions of CHBA can fit the density map (Fig C and D).

(a) To determine the correct binding pose of CHBA, we performed a detailed interaction analysis and found that the CHBA-forward direction could form additional hydrogen bonds with the $E166^{45.51}$ and $R71^{2.60}$ than the CHBA-reverse direction, which meant a more stable binding pose (Fig E and I).

(b) Moreover, the molecular dynamics simulations also confirmed that the forward binding conformation of CHBA was more stable (Fig C and E). In the simulation trajectories initiated from the forward CHBA structure, the RMSD of CHBA remained around 1.2 Å, and the five representative conformations were essentially the same as the initial structure (Fig F-H). In contrast, for the trajectories initiated from the reverse CHBA structure, the RMSD distribution was more dispersed and exhibited larger values, ranging from 2.5 to 6 Å, with significant deviations observed between the five representative conformations and the initial structure (Fig J and L).

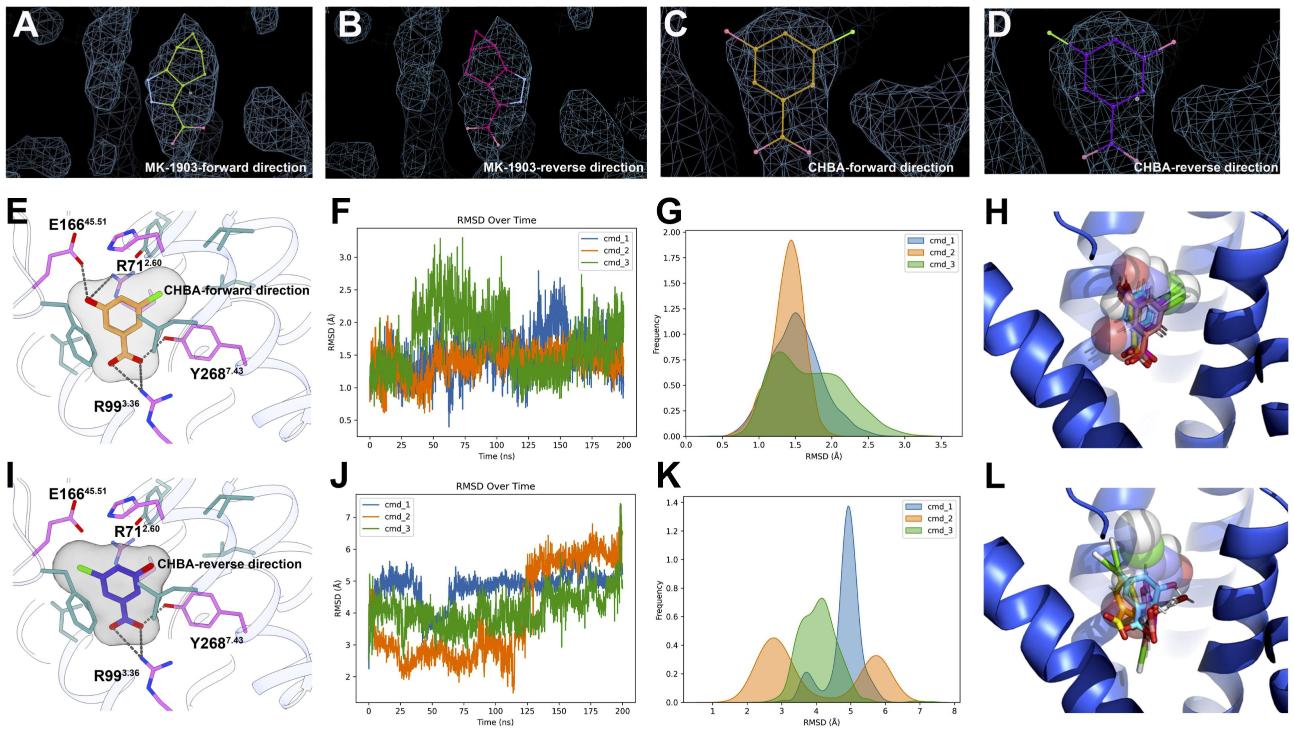

Fig. Analysis of the ligand binding orientation. (A-D) The forward and reverse directions of MK-1903 and CHBA. **(E, I)** Detailed interaction analysis of CHBA in the forward and reverse directions. **(F)** Change of RMSD over time for CHBA in three parallel trajectories initiated from the forward CHBA structure. **(G)** Probability density distribution of RMSD for the forward CHBA structure. **(H)** Five representative structures from trajectories initiated with the forward CHBA structure, with the initial structure shown as sphere. **(J)** Change of RMSD over time for CHBA in three parallel trajectories initiated from the reverse CHBA structure. **(K)** Probability density distribution of RMSD for the reverse CHBA structure. **(L)** Five representative structures from trajectories initiated with the reverse CHBA structure, with the initial structure shown as sphere.

Question 2. The cryo-EM density of critical residues, particularly those examined in the mutagenesis studies, should be shown to provide evidence for the accuracy of the structural modeling.

Answer 2. Thank you very much for your kind and professional comments and suggestions. According to your requirement, the cryo-EM densities of critical residues, particularly those examined in the mutagenesis studies, have been shown in Figs S6A-S6C, S12E and S12F.

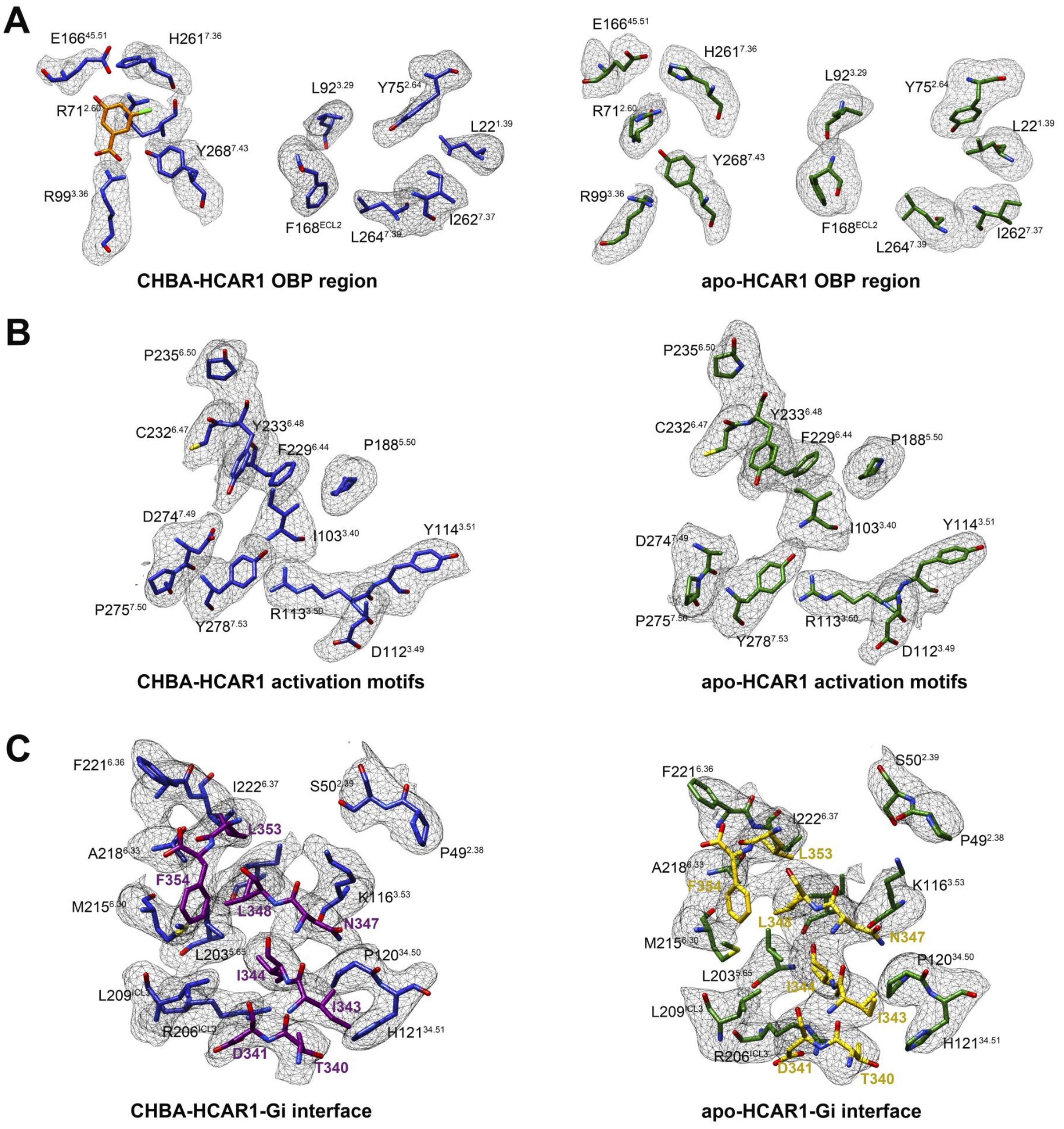

S6 Fig. Density maps of critical residues in the apo and CHBA-bound HCAR1. (A) Density maps of hydrophilic and hydrophobic residues in the OBP region. (B) Density maps of key activation motifs. (C) Density maps of key residues in the HCAR1-Gi interface. Royal blue-dark magenta, CHBA-HCAR1-Gi1; forest green-beige, apo-HCAR1-Gi1; orange, CHBA.

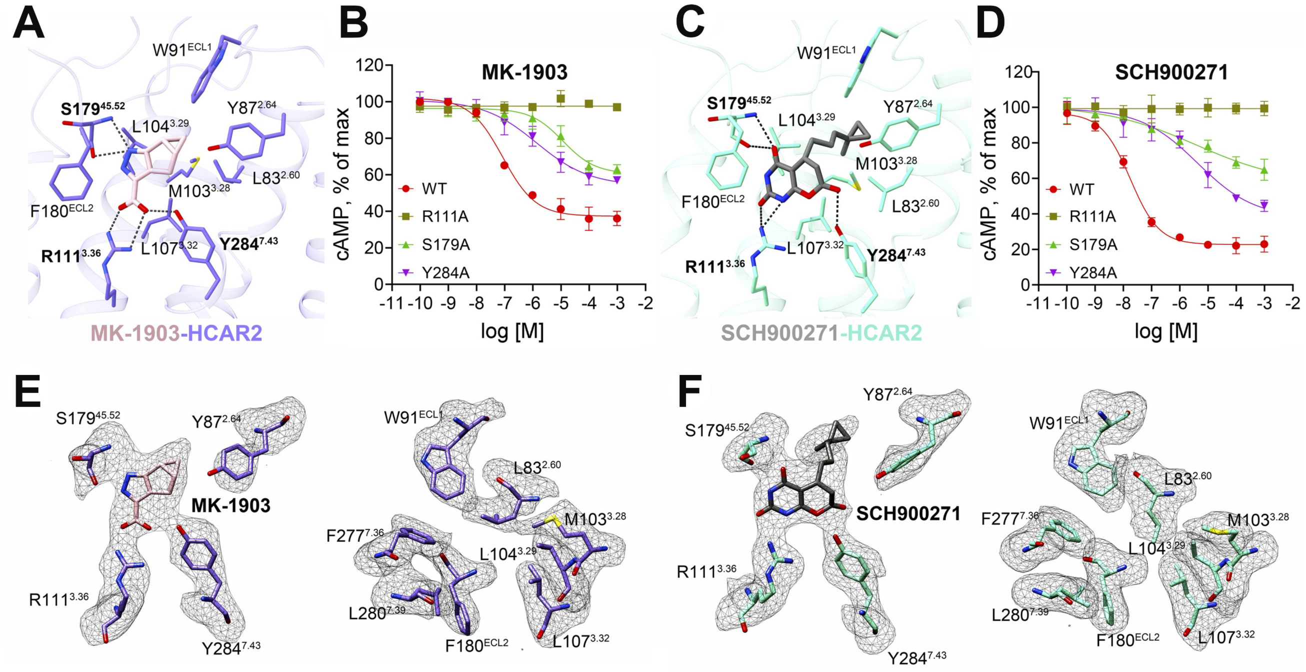

S12 Fig. Interaction analysis of MK-1903 and SCH900271 with HCAR2 in the OBP region. (A, B) Specific interactions of MK-1903 with HCAR2. Effects on Gi-mediated cAMP by single point mutations of key residues that interact with MK-1903. **(C, D)** Specific interactions of SCH900271 with HCAR2. Effects on Gi-mediated cAMP by single point mutations of key residues that interact with SCH900271. The data are presented as means ± SEM. The experiments are performed in triplicate. **(E, F)** Density maps of MK-1903, SCH900271, and surrounding key residues. The ligands and residues are shown using a stick representation. Pink-medium slate blue, MK-1903-HCAR2; gray-aquamarine, SCH900271-HCAR2.

Question 3. The authors state that HCAR1 exhibits high basal activity based on the fact that they could assemble the ligand-free complex with Gi in their structural studies. However, this is insufficient. Experimental data are required to directly demonstrate the basal activity of HCAR1.

Answer 3. Thank you very much for your kind and professional comments and suggestions. The existence of the apo state of HCAR1-Gi1 complex implied that HCAR1 did not necessarily require ligand binding to activate the downstream signaling transducers. In fact, a self-activated state was

also observed in the subfamily receptor HCAR2 (PDB: 8IJ3) and HCAR3 (PDB: 9JIC). In order to demonstrate the basal activity of HCAR1, the cAMP accumulation assay was conducted. The results showed that in the absence of a ligand, the downstream cAMP level decreased as the concentration of the HCAR1 receptor increased, which further corroborated the basal activity of HCAR1. We have added the related results and figure in the manuscript. (Please see line 286-288, Fig. S15B)

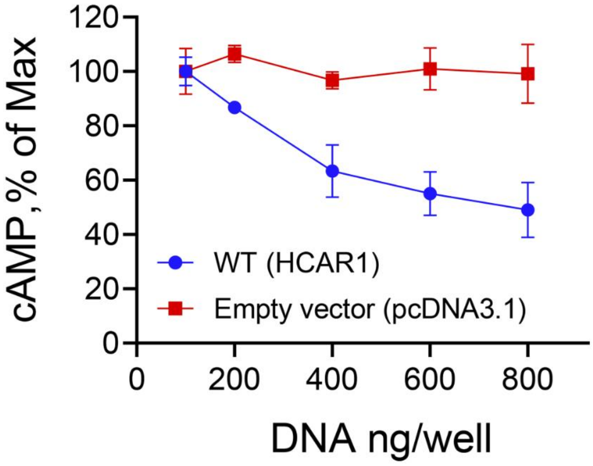

S15 Fig. (B) Effects of increasing HCAR1 receptor concentration on Gi-mediated cAMP.

Question 4. The authors suggest that the different conformations of ECL2 in HCAR1 and HCAR2 partly account for ligand selectivity based on their structural comparison analysis. However, these differences might be artifacts of cryo-EM modeling, considering the low resolution of the receptor extracellular regions. Conducting additional MD simulations would be valuable to explore the conformational dynamics of ECL2 in these two receptors when bound to subtype-selective agonists.

Answer 4. Thank you very much for your kind and professional comments and suggestions. The ECL2 regions of HCAR1 and HCAR2 showed marked differences in spatial position. Particularly, the Cα atom of $S167^{45.52}$ in HCAR1 moved about 3.3 Å relative to $S179^{45.52}$ in HCAR2. Meanwhile, the key residue $E166^{45.51}$ in the ECL2 of HCAR1, which interacted with CHBA, was replaced by $S178^{45.51}$ in HCAR2.

(a) To explore the role of ECL2 in agonist selectivity between HCAR1 and HCAR2, molecular dynamics simulations were conducted (S8A and S8B Fig). After performing 200 ns of MD

simulations on CHBA-bound HCAR1 and MK1903-bound HCAR2, it was found that the RMSD values of the ECL2 regions for both complexes exceeded 10 Å, which indicated a high degree of flexibility in this loop region (S8C–8F Fig). Furthermore, interaction analysis revealed that CHBA primarily formed hydrogen bonds with the carboxyl group of $E166^{45.51}$ in HCAR1 (S8G Fig). In contrast, MK1903 formed stable hydrogen bonds with the side chain and backbone of $S179^{45.52}$ in HCAR2 (S8H Fig). We considered that the differences in the interactions between ligands and ECL2 might be a crucial factor influencing agonist selectivity between HCAR1 and HCAR2.

(b) Molecular dynamics simulation methods. The Membrane Builder module in CHARMM-GUI server was used to prepare the simulation inputs, including a membrane of pre-equilibrated (310 K) POPC lipids based on the OPM database alignment, TIP3P solvent with 0.15 M Na+/Cl- ions, and the CHARMM36 forcefield. The force field of the ligands was generated by the CGenFF program. All MD simulations were performed using GROMACS-2019.4. The CHARMM36m forcefield was used to describe the interactions in the system. Energy minimization was performed for 5,000 steps by the steepest descent algorithm. Then a 250 ps NVT simulation was performed at 310 K for solvent equilibration, followed by a 1.6 ns NPT equilibration to 1 atm using the Parrinello-Rahman barostat. All MD simulations were performed with a time-step of 2 fs. Long-range electrostatic interactions were treated by the particle-mesh Ewald method. The short-range electrostatic and van der Waals interactions both used a cutoff of 10 Å. All bonds were constrained by the LINCS algorithm. Here, MD simulations were started from the solved structures of the MK1903-bound HCAR2 and the CHBA-bound HCAR1. Simulation runs for 200 ns. The trajectory was analyzed by the python package MDAnalysis. We have added the related results, methods and figure in the manuscript. (Please see line 191-203, 534-548, Fig. S8)

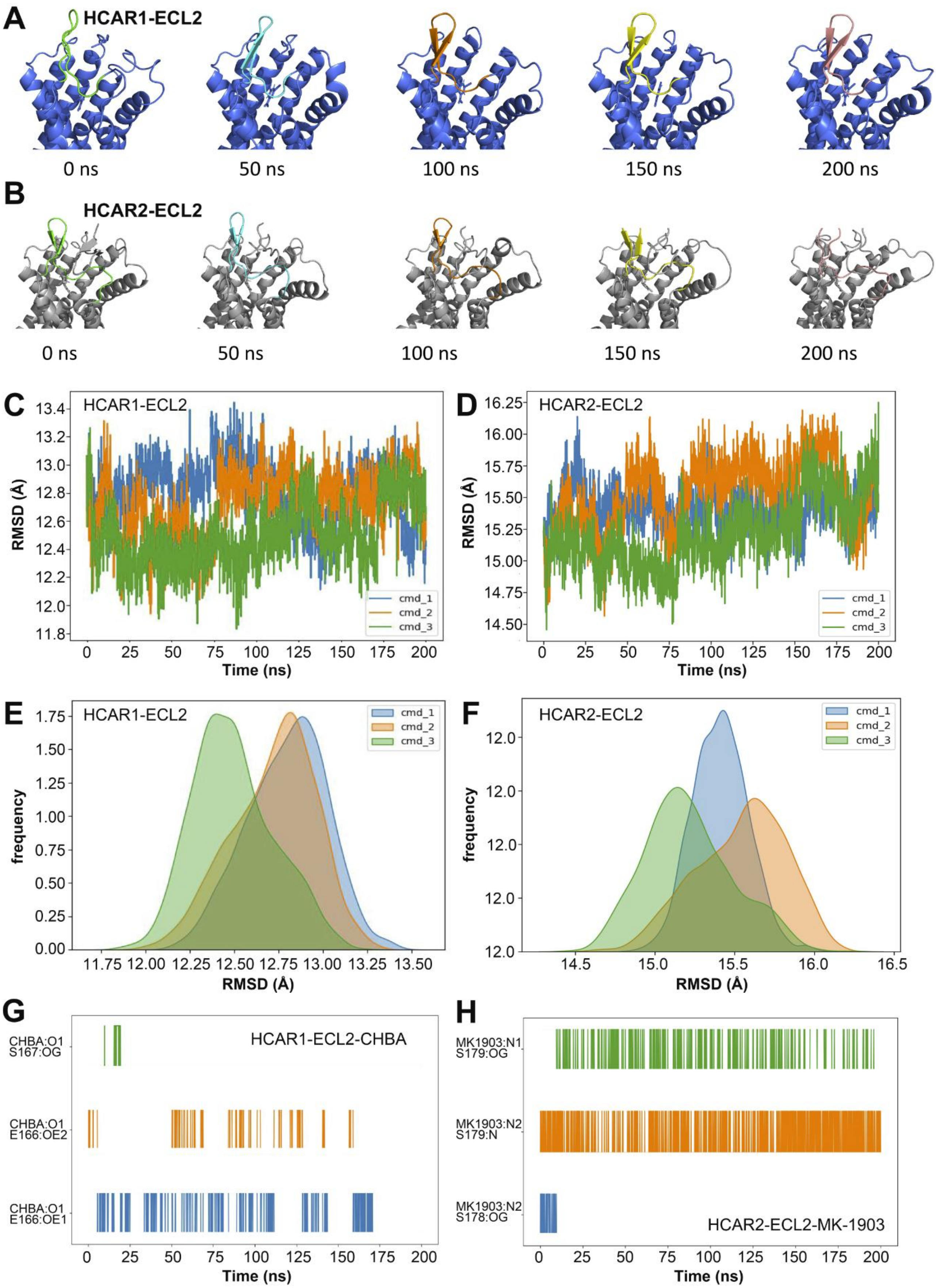

A
HCAR1-ECL2
0 ns
50 ns
100 ns
150 ns
200 ns
B
HCAR2-ECL2
0 ns
50 ns
100 ns
150 ns
200 ns
C
HCAR1-ECL2
RMSD (Å)
Time (ns)
cmd_1
cmd_2
cmd_3
D
HCAR2-ECL2
RMSD (Å)
Time (ns)
cmd_1
cmd_2
cmd_3
E
HCAR1-ECL2
frequency
RMSD (Å)
cmd_1
cmd_2
cmd_3
F
HCAR2-ECL2
frequency
RMSD (Å)
cmd_1
cmd_2
cmd_3
G
HCAR1-ECL2-CHBA
CHBA:O1
S167:OG
CHBA:O1
E166:OE2
CHBA:O1
E166:OE1
Time (ns)
H
MK1903:N1
S179:OG
MK1903:N2
S179:N
MK1903:N2
S178:OG
HCAR2-ECL2-MK-1903
Time (ns)

S8 Fig. Different interaction patterns of ECL2 with the agonist in HCAR2 and HCAR1. Conformational changes of ECL2 in the simulation trajectories of the HCAR1 (A) and HCAR2 (B) complexes at 0 ns, 50 ns, 100 ns, 150 ns, and 200 ns. RMSD of the ECL2 backbone atoms in the HCAR1 (C, E) and HCAR2 (D, F) complexes. The hydrogen bonds between ECL2 and ligand during the trajectories in HCAR1 (G) and HCAR2 (H) complexes are shown. Royal blue, HCAR1; light gray, HCAR2.

Question 5. Some of the content in the Introduction and Discussion regarding the functional roles of HCAR1 is redundant. Streamlining the Discussion section is recommended.

Answer 5. Thank you very much for your valuable suggestion. According to your requirement, we have streamlined the Discussion section. In particular, the content in the Discussion regarding the functional roles of HCAR1 has been deleted. (Please see line 352-362)

Question 6. It could be informative to compare the structures of HCAR1 and HCAR2 with those of short-chain fatty acid receptors FFA2 and FFA3, as their endogenous ligands share a high degree of chemical similarity.

Answer 6. Thank you very much for your kind and professional comments and suggestions. The HCAR family comprises three members: HCAR1, HCAR2 (GPR109A), and HCAR3 (GPR109B). Their endogenous ligands are lactate, β-OHB, and 3-hydroxyoctanoic acid, respectively. Interestingly, the endogenous ligands of free fatty acid receptors 2 and 3 (FFAR2/3) are short-chain fatty acids as well. Of these, FFAR2 is predominantly activated by acetate (AA), butyrate (BA), and propionate, while FFAR3 is activated by BA, propionate, valerate, and caproate. Structural comparisons revealed that the positions of agonists in HCAR1/2 and FFAR2/3 were entirely different (S17A Fig). In HCAR1/2, the agonists CHBA and β-OHB were located within the orthosteric pocket formed by TM1, TM2, TM3, TM7, and ECL2. In FFAR2/3, the agonists AA and

BA were situated in a pocket composed of TM3, TM4, TM5, TM6, and TM7. Further analysis suggested that despite the significant chemical similarity shared by the endogenous ligands of the HCAR and FFAR families, the key amino acids responsible for recognizing the acidic group of agonists were not conserved (S17B–17G Fig). Specifically, the $Arg^{3.36}$ was regarded as a critical determinant for the recognition of agonist acidic group in HCAR1/2, while the residue was substituted with $Ile^{3.36}$ in FFAR2/3 (S17E Fig). In contrast, the carboxyl group of AA and BA formed salt bridges with several basic residues $Arg^{5.39}$, $His^{6.55}$ and $Arg^{7.35}$ in FFAR2/3, whereas these residues were replaced by $His^{5.39}$, $Arg^{6.55}$ and $Leu/Phe^{7.35}$ in HCAR1/2 (S17F Fig). We speculated that these differences in key amino acids might ultimately result in distinct ligand binding modes in the HCAR and FFAR families.

We have added the related results and figure in the manuscript. (Please see line 369-386, Fig. S17)

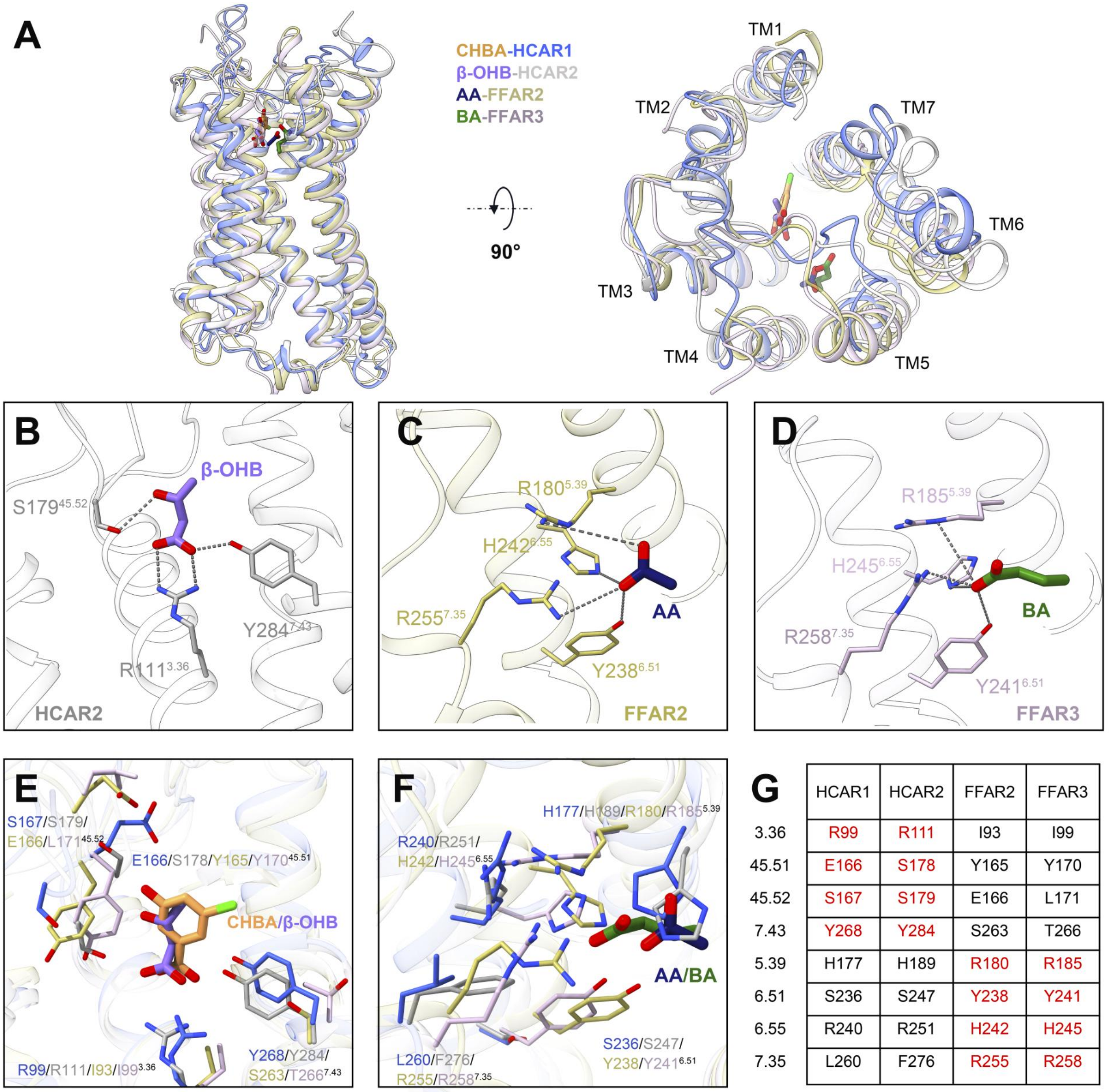

	HCAR1	HCAR2	FFAR2	FFAR3
3.36	R99	R111	I93	I99
45.51	E166	S178	Y165	Y170
45.52	S167	S179	E166	L171
7.43	Y268	Y284	S263	T266
5.39	H177	H189	R180	R185
6.51	S236	S247	Y238	Y241
6.55	R240	R251	H242	H245
7.35	L260	F276	R255	R258

S17 Fig. Structural comparison between CHBA-HCAR1, β-OHB-HCAR2 (PDB: 8J6Q), AA-FFAR2 (PDB: 8J24), and BA-FFAR3 (PDB: 8J21). (A) Superposition of the architecture of CHBA-HCAR1, β-OHB-HCAR2, AA-FFAR2, and BA-FFAR3. **(B)** Detailed polar interactions of β-OHB with HCAR2. **(C)** Detailed polar interactions of AA with FFAR2. **(D)** Detailed polar interactions of BA with FFAR3. **(E)** Comparison of the key amino acids responsible for ligand recognition in the OBP region of HCAR1 and HCAR2. **(F)** Comparison of the key amino acids responsible for ligand recognition in the OBP region of FFAR2 and FFAR3. **(G)** Sequence alignment of key amino acids in HCAR1/2 and FFAR2/3. Orange-royal blue, CHBA-HCAR1; medium purple-light gray, β-OHB-HCAR2; dark blue-dark khaki, AA-FFAR2; dark green-thistle, BA-FFAR3.

Response to Reviewer 3

Question 1. Figure 1. It is understandable that the GPCR field is very much used to cryo-EM structures and without any labelling one can recognise the receptor and the G protein - however to a reader outside of the field it would be helpful to see which part is in the membrane, which part is peripheral to the membrane, etc.

Answer 1. Thank you very much for your valuable suggestion. According to your requirement, we have revised Figure 1. This allows for a clearer display of which parts are inside the membrane and which parts are outside the membrane. (Please see Fig. 1)

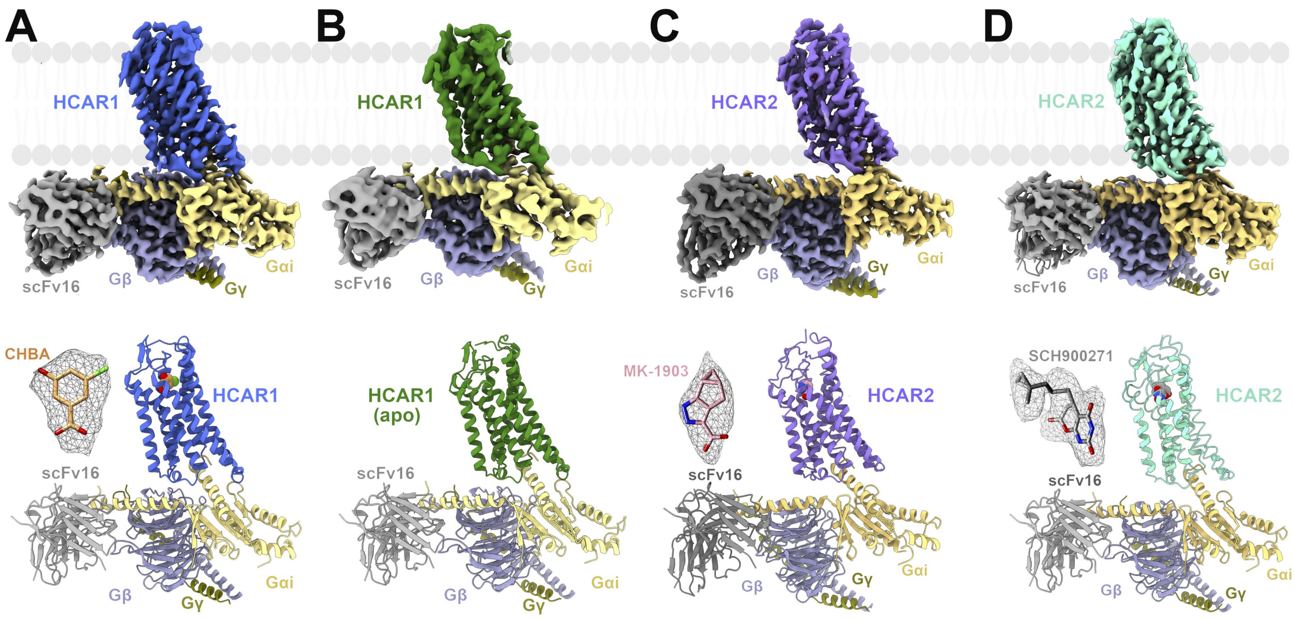

Fig 1. Cryo-EM structures of HCAR1-Gi1 and HCAR2-Gi1 complexes. (A−D) Cryo-EM map and structural model of HCAR1-Gi1-scFv16 complex in the presence of CHBA (**A**) and apo form (**B**). Cryo-EM maps and structural models of HCAR2-Gi1-scFv16 complex in the presence of MK-1903 (**C**) and SCH900271 (**D**). The density of the agonist (shown as sticks) is depicted as gray meshes. The maps and structural models are colored by subunits. Royal blue, CHBA-HCAR1; forest green, apo-HCAR1; medium slate blue, MK-1903-HCAR2; aquamarine, SCH900271-HCAR2; light yellow, Gαi; slate blue, Gβ; olive, Gγ; gray, scFv16; orange, CHBA; pink, MK-1903; gray, SCH900271.

Question 2. Is the ligand binding site in the apo HCAR1 occupied by any density?

Answer 2. Thank you very much for your kind and professional comments and suggestions. When we compared the densities of apo and CHBA-bound HCAR1 from the same perspective, we noticed that there was no observable density at the ligand binding site of apo HCAR1.

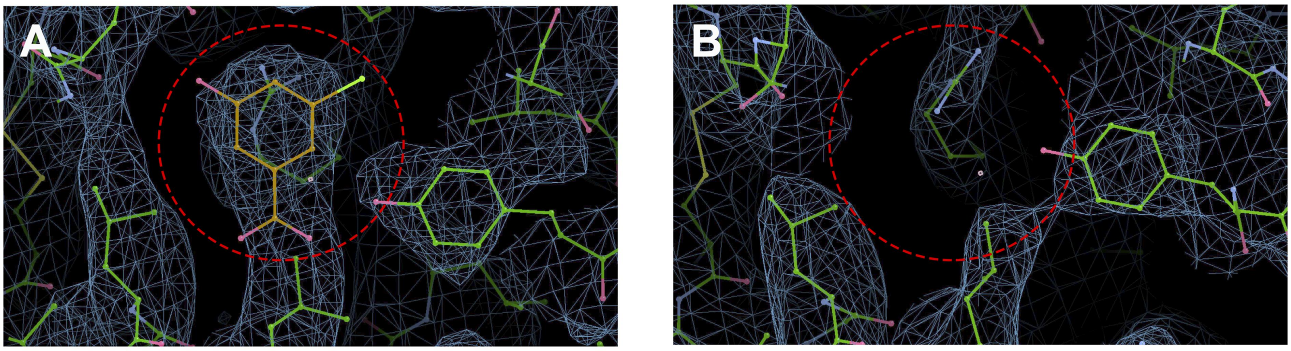

CHBA-bound HCAR1 **apo HCAR1**

Fig. Densities of apo and CHBA-bound HCAR1 from the same perspective.

Question 3. Figure 4c-d. The figure panels seem to be very busy and thus difficult to understand. It could be that this impression comes from the multiple labels crammed into the figure panels. One possible way to deal with this could be to offload the compound colours to panel 4a (e.g., write compound names in the colour that is used in the c and d). But apart from this, there are many elements shown in these multiple comparison panels (c-d), which makes this figure rather difficult to follow. Some simplification would be useful.

The supplementary figure 13 features similar comparisons, but there the presentation is easier on the eye.

Answer 3. Thank you very much for your kind and professional comments and suggestions. According to your requirement, we have simplified and adjusted Figure 4c-d to make the figure look clearer. (Please see Fig. 4)

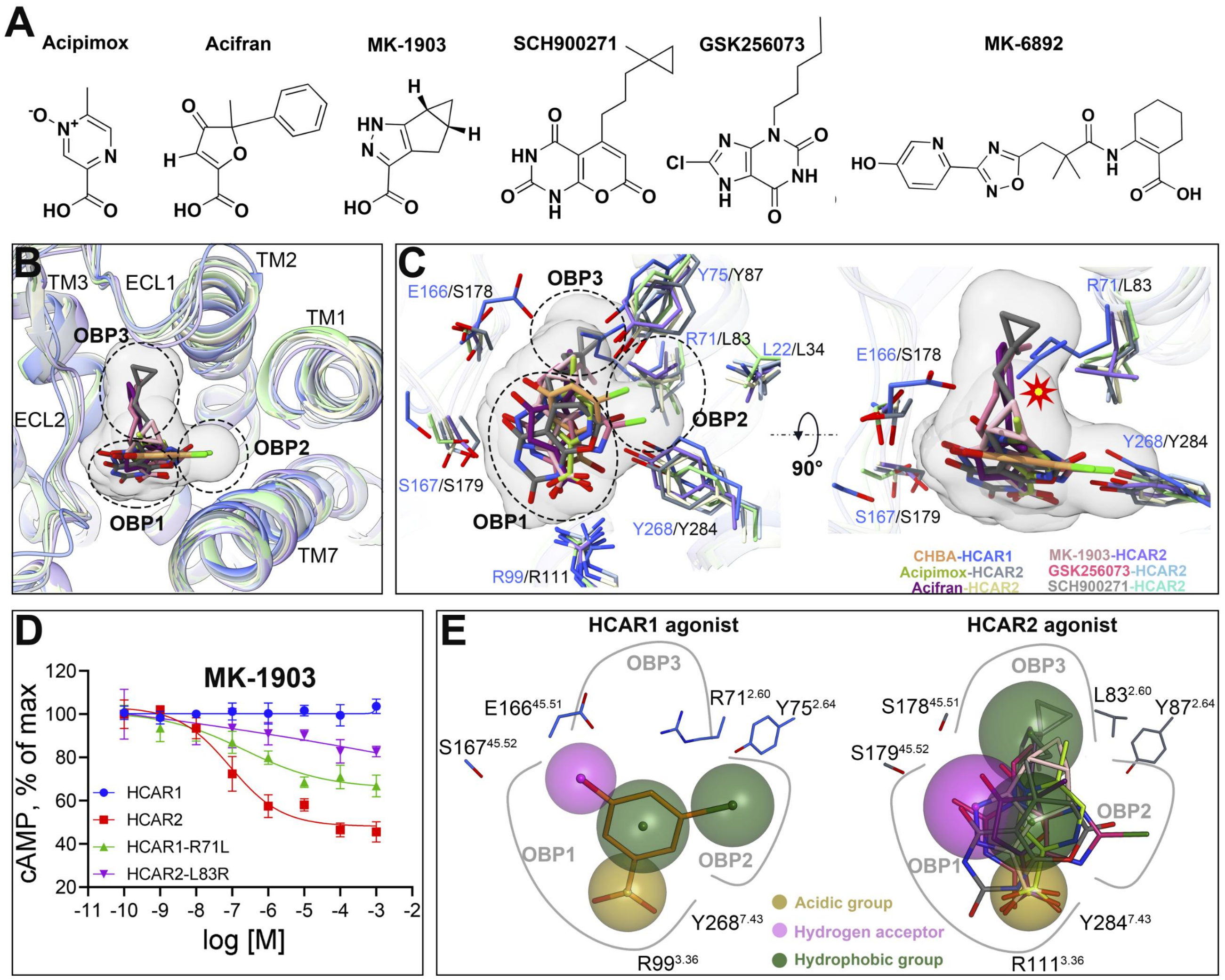

Fig 4. Structural features of agonists that match the HCAR1 and HCAR2 orthosteric pocket. **(A)** Chemical structures of the HCAR2 subtype-specific agonists acipimox, acifran, MK-1903, SCH900271, GSK256073, and MK-6892. **(B)** Superposition of the orthosteric agonists of HCAR1 and HCAR2, including CHBA, acipimox (PDB: 8IJB), acifran (PDB: 8IHI), GSK256073 (PDB: 8I7W), MK-1903, and SCH900271. **(C)** Comparison of different ligand binding modes of HCAR1 and HCAR2 in the OBP regions. Orange-royal blue, CHBA-HCAR1; yellow green-slate gray, acipimox-HCAR2; pink-medium slate blue, MK-1903-HCAR2; purple-lemon chiffon, acifran-HCAR2; rose red-light sky blue, GSK256073-HCAR2; gray-aquamarine, SCH900271-HCAR2. **(D)** Effects on Gi-mediated cAMP by single point mutations of R71$^{2.60}$L in HCAR1 and L83$^{2.60}$R in HCAR2. The data are presented as means ± SEM. The experiments are performed in triplicate. **(E)** Structural features that differentiate HCAR1 and HCAR2 agonists. Dark goldenrod, acidic group; magenta, hydrogen bond acceptor; dark green, hydrophobic group.

Question 4. Figure 4f. Here the colours are partially overlapping with c-d, but now describing properties of the atoms. This can be somewhat confusing to the reader.

Answer 4. Thank you very much for your valuable suggestion. According to your requirement, we have changed the color of Figure 4F (now Figure 4E) to avoid color overlap with that of Figure 4C. (Please see Fig. 4)

Question 5. Figure 6 and the corresponding description. It is not clear that this part of the manuscript advances our understanding of Gi coupling. The authors just describe the interfacial interactions, stating the obvious facts. My impression is that it could be a figure that could be moved to the supplementary.

Answer 5. Thank you very much for your kind and professional comments and suggestions. Our previous results indeed had insufficient analysis of Gi coupling. Therefore, to enhance understanding, we conducted a more in-depth analysis of Gi coupling. Previous studies have emphasized the critical roles of the ICL2 and ICL3 regions in G protein coupling. In the case of HCAR1–3 receptors, sequence analysis revealed that despite the presence of two distinct residues in the ICL2 region, their three-dimensional structures overlapped well (S16A–16F Fig). In contrast, the ICL3 region showed a clear sequence divergence: (1) HCAR1 contained one additional residue ($Q208^{ICL3}$) compared to HCAR2/3. (2) Three residues in HCAR1, namely $L209^{ICL3}$, $A210^{ICL3}$, and $Q212^{ICL3}$, were substituted with $M220^{ICL3}$, $D221^{ICL3}$, and $H223^{ICL3}$ in HCAR2/3 (S16G–16J Fig). Consequently, a notable conformational difference was observed in the ICL3 region between HCAR1 and HCAR2/3, which might explain the different shifts of the α5 and αN helices (S16B Fig). Overall, our findings clarified the Gi1 coupling features of HCAR1 and enhanced the understanding of the G protein coupling mechanism.

We have added the related results and figure in the manuscript. (Please see line 340-349, Fig. S16)

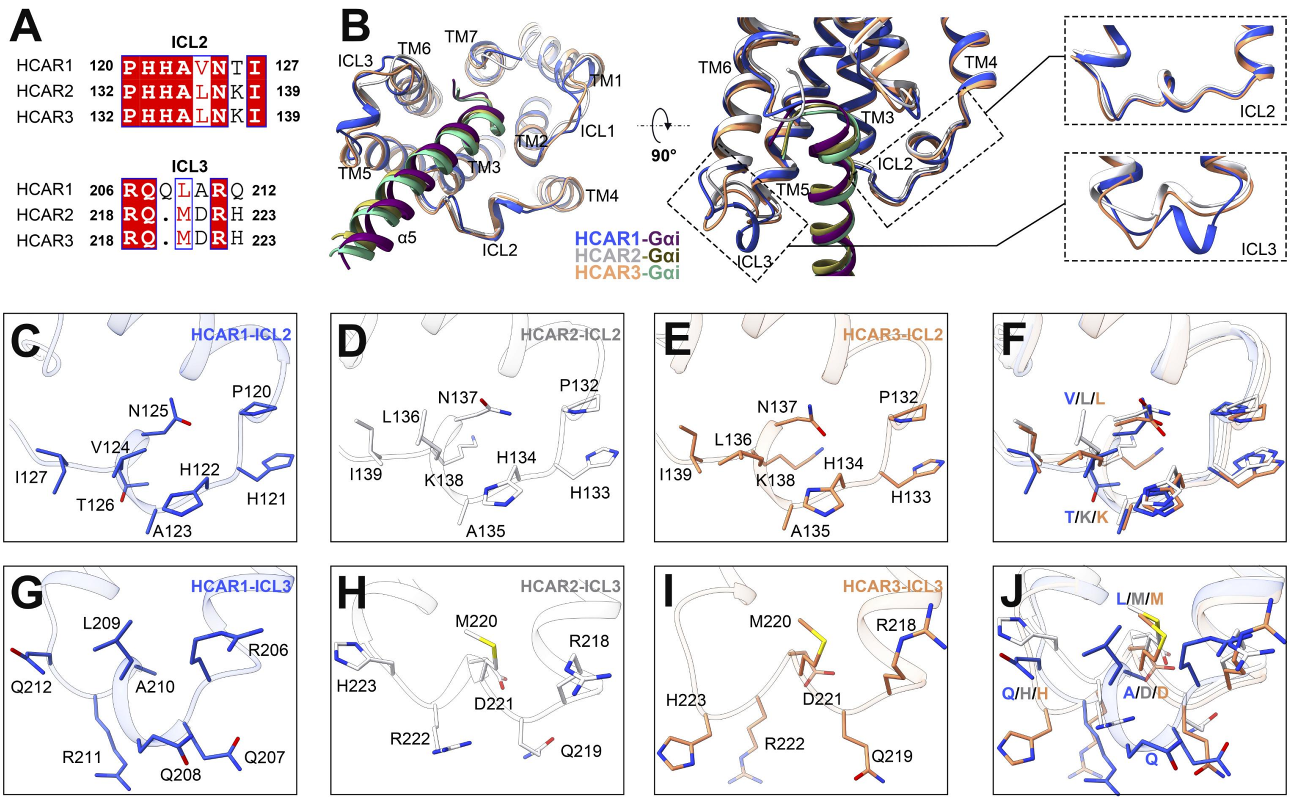

S16 Fig. Comparison of the HCAR1-Gi1 with HCAR2-Gi1 and HCAR3-Gi1. (A) Sequence alignment of ICL2 and ICL3 regions in HCAR1–3. Positions that are identical between the receptors are highlighted with a red background. **(B)** Superposition of the architecture of ICL2 and ICL3 regions in HCAR1–3. **(C−F)** Detailed distribution of amino acids in the ICL2 of HCAR1–3. **(G−J)** Detailed distribution of amino acids in the ICL3 of HCAR1–3. Royal blue-dark magenta, CHBA-HCAR1-Gi1; light gray-dark khaki, HCAR2-Gi1; sandy brown-medium aquamarine, HCAR3-Gi1.

Question 6. A figure that describes the key findings and mechanistic insights that the authors could derive from their analysis is currently missing. This would be a very useful illustration that would visually outline the main messages of this manuscript. I think such an illustration would allow the authors to end the manuscript with a powerful statement (if they manage to do it). Such a figure could work well with a revised Discussion.

Answer 6. Thank you very much for your kind and professional comments and suggestions. This study mainly showcases the HCAR1-Gi1 complexes in both the agonist-bound and apo forms, and the HCAR2-Gi1 complexes in the agonist-bound form.

(a) Studies of ligand recognition mechanisms suggested that the orthosteric agonist CHBA bound to HCAR1 by directly interacting with four key residues, $R99^{3.36}$, $Y268^{7.43}$, $E166^{45.51}$, and $R71^{2.60}$, in the OBP. Notably, a comparison of the apo and CHBA-bound HCAR1 structures suggested that the addition of the ligand induced a large conformational change in the side chain of $R99^{3.36}$, thus facilitating the formation of a salt bridge with the carboxyl group of CHBA.

(b) Regarding the mechanism of agonist selectivity, our results suggested that three residues in HCAR1/HCAR2, $R79/W91^{ECL1}$, $Y75/Y87^{2.64}$, and $R71/L83^{2.60}$, played a decisive role. For the HCAR1 subtype-specific agonist CHBA, the bulky residue $W91^{ECL1}$ in HCAR2 led to $Y87^{2.64}$ shifting too close to the 5-position chloro group of CHBA relative to $Y75^{2.64}$ in HCAR1, thus generating steric hindrance effect. Consequently, CHBA selectively activated HCAR1 but not HCAR2. For many HCAR2 subtype-specific agonists, such as acipimox, MK-1903, acifran, GSK256073, and SCH900271, they all contained a small or large hydrophobic group that inserted into the OBP3 region of HCAR2. In contrast, the OBP3 entrance of HCAR1 was occluded by the side chain of $R71^{2.60}$. Thus, significant steric hindrance precluded these HCAR2-specific agonists from binding to HCAR1.

According to your requirement, we have added an illustration to outline the main messages of this manuscript. (Please see line 356-362, 387-391, 395-399, Fig. 7)

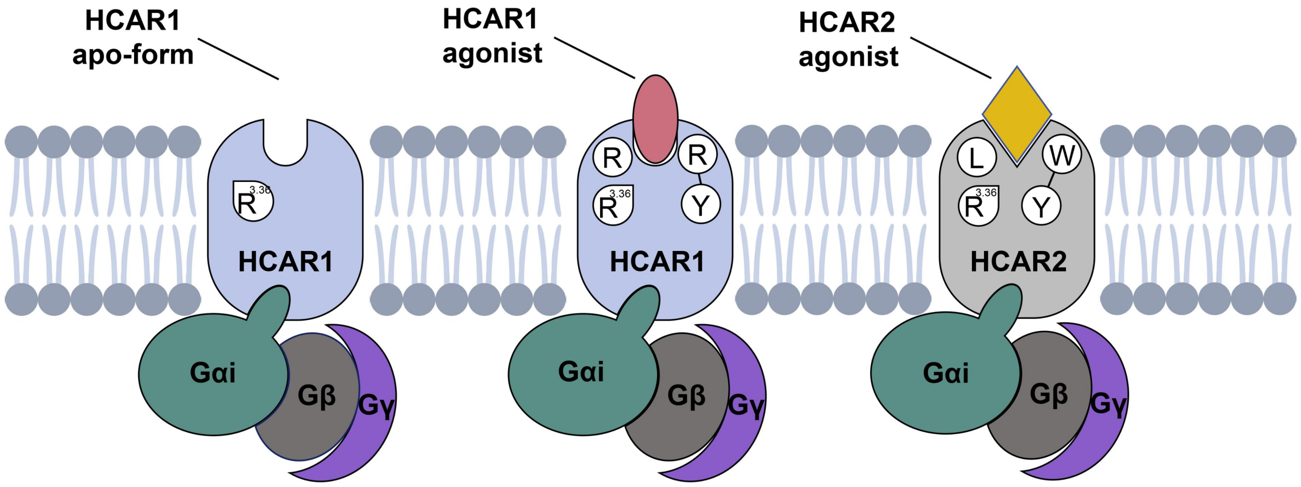

Fig 7. Mechanism diagram of ligand recognition and selectivity of HCAR1. This study mainly showcases the HCAR1-Gi1 complexes in both the agonist-bound and apo forms, and the HCAR2-Gi1 complexes in the agonist-bound form. Four residues of HCAR1, namely R99$^{3.36}$, Y268$^{7.43}$, E166$^{45.51}$, and R71$^{2.60}$, are deemed crucial for ligand recognition. Notably, R99$^{3.36}$ undergoes a significant conformational change upon agonist binding. Furthermore, three residues in HCAR1/HCAR2, namely R71/L83$^{2.60}$, R79/W91^{ECL1}, and Y75/Y87$^{2.64}$, play a decisive role in agonist selectivity between HCAR1 and HCAR2. Light blue, HCAR1; light gray, HCAR2; light coral, HCAR1 agonist; gold, HCAR2 agonist; dark cyan, Gαi; dark gray, Gβ; dark orchid, Gγ.

Question 7. I would recommend to revise the Discussion by removing the redundant paragraph 1 (or fusing it with the introduction) - and adding the figure (see point 5 above) that would help visualise the key findings mentioned in the discussion. The Discussion part could also be a good opportunity to look more broadly at the findings in the context of the whole GPCR/Gi field, not just repeating what was already said in the results and not focusing exclusively on HCARs.

Answer 7. Thank you very much for your kind and professional comments and suggestions.

(a) According to your requirement, we have streamlined the Discussion section. In particular, the redundant paragraph 1 has been deleted.

(b) We have added the figure 7 to help visualise the key findings mentioned in the discussion. (Please see line 356-362, 387-391, 395-399, Fig. 7)

(c) We have added a comparison about the structures of HCAR1 and HCAR2 with those of short-chain fatty acid receptors FFAR2 and FFAR3 in the discussion. This is because their endogenous ligands share a high degree of chemical similarity.

The HCAR family comprises three members: HCAR1, HCAR2 (GPR109A), and HCAR3 (GPR109B). Their endogenous ligands are lactate, β-OHB, and 3-hydroxyoctanoic acid, respectively. Interestingly, the endogenous ligands of free fatty acid receptors 2 and 3 (FFAR2/3) are short-chain fatty acids as well. Of these, FFAR2 is predominantly activated by acetate (AA), butyrate (BA), and propionate, while FFAR3 is activated by BA, propionate, valerate, and caproate. Structural comparisons revealed that the positions of agonists in HCAR1/2 and FFAR2/3 were entirely different (S17A Fig). In HCAR1/2, the agonists CHBA and β-OHB were located within the orthosteric pocket formed by TM1, TM2, TM3, TM7, and ECL2. In FFAR2/3, the agonists AA and BA were situated in a pocket composed of TM3, TM4, TM5, TM6, and TM7. Further analysis suggested that despite the significant chemical similarity shared by the endogenous ligands of the HCAR and FFAR families, the key amino acids responsible for recognizing the acidic group of agonists were not conserved (S17B–17G Fig). Specifically, the $Arg^{3.36}$ was regarded as a critical determinant for the recognition of agonist acidic group in HCAR1/2, while the residue was substituted with $Ile^{3.36}$ in FFAR2/3 (S17E Fig). In contrast, the carboxyl group of AA and BA formed salt bridges with several basic residues $Arg^{5.39}$, $His^{6.55}$ and $Arg^{7.35}$ in FFAR2/3, whereas these residues were replaced by $His^{5.39}$, $Arg^{6.55}$ and $Leu/Phe^{7.35}$ in HCAR1/2 (S17F Fig). We speculated that these differences in key amino acids might ultimately result in distinct ligand binding modes in the HCAR and FFAR families.

We have added the related results and figure in the discussion. (Please see line 369-386, Fig. S17)

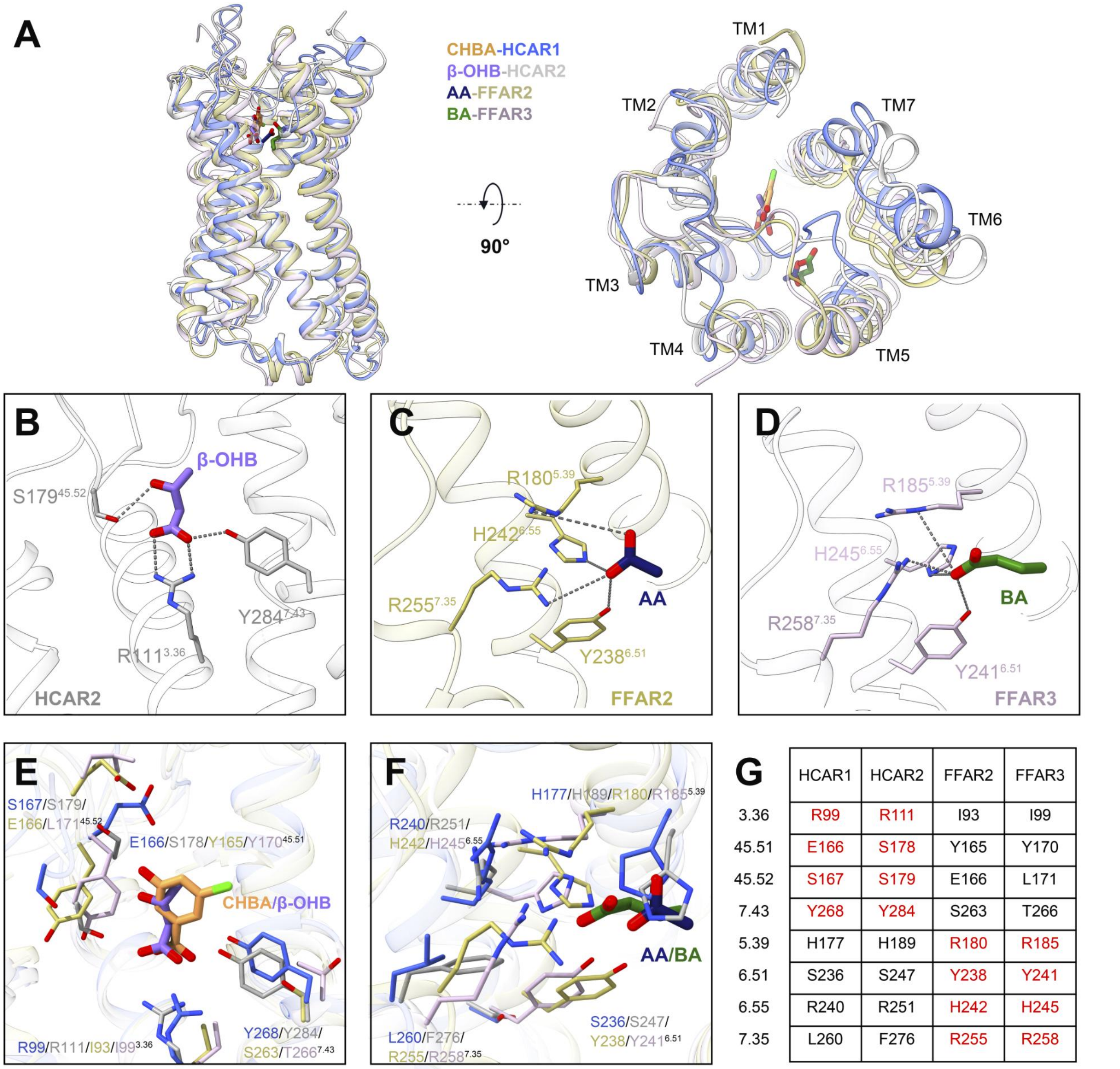

	HCAR1	HCAR2	FFAR2	FFAR3
3.36	R99	R111	I93	I99
45.51	E166	S178	Y165	Y170
45.52	S167	S179	E166	L171
7.43	Y268	Y284	S263	T266
5.39	H177	H189	R180	R185
6.51	S236	S247	Y238	Y241
6.55	R240	R251	H242	H245
7.35	L260	F276	R255	R258

S17 Fig. Structural comparison between CHBA-HCAR1, β-OHB-HCAR2 (PDB: 8J6Q), AA-FFAR2 (PDB: 8J24), and BA-FFAR3 (PDB: 8J21). (A) Superposition of the architecture of CHBA-HCAR1, β-OHB-HCAR2, AA-FFAR2, and BA-FFAR3. **(B)** Detailed polar interactions of β-OHB with HCAR2. **(C)** Detailed polar interactions of AA with FFAR2. **(D)** Detailed polar interactions of BA with FFAR3. **(E)** Comparison of the key amino acids responsible for ligand recognition in the OBP region of HCAR1 and HCAR2. **(F)** Comparison of the key amino acids responsible for ligand recognition in the OBP region of FFAR2 and FFAR3. **(G)** Sequence alignment of key amino acids in HCAR1/2 and FFAR2/3. Orange-royal blue, CHBA-HCAR1; medium purple-light gray, β-OHB-HCAR2; dark blue-dark khaki, AA-FFAR2; dark green-thistle, BA-FFAR3.

Question 8. Supplementary figure 6 should show the densitiy of the ligand (CHBA), not only the residues. Similar to supplementary figures 11e-f.

Answer 8. According to your requirement, the cryo-EM densities of critical residues and ligand (CHBA) in HCAR1 have been added. (Please see Fig. S6)

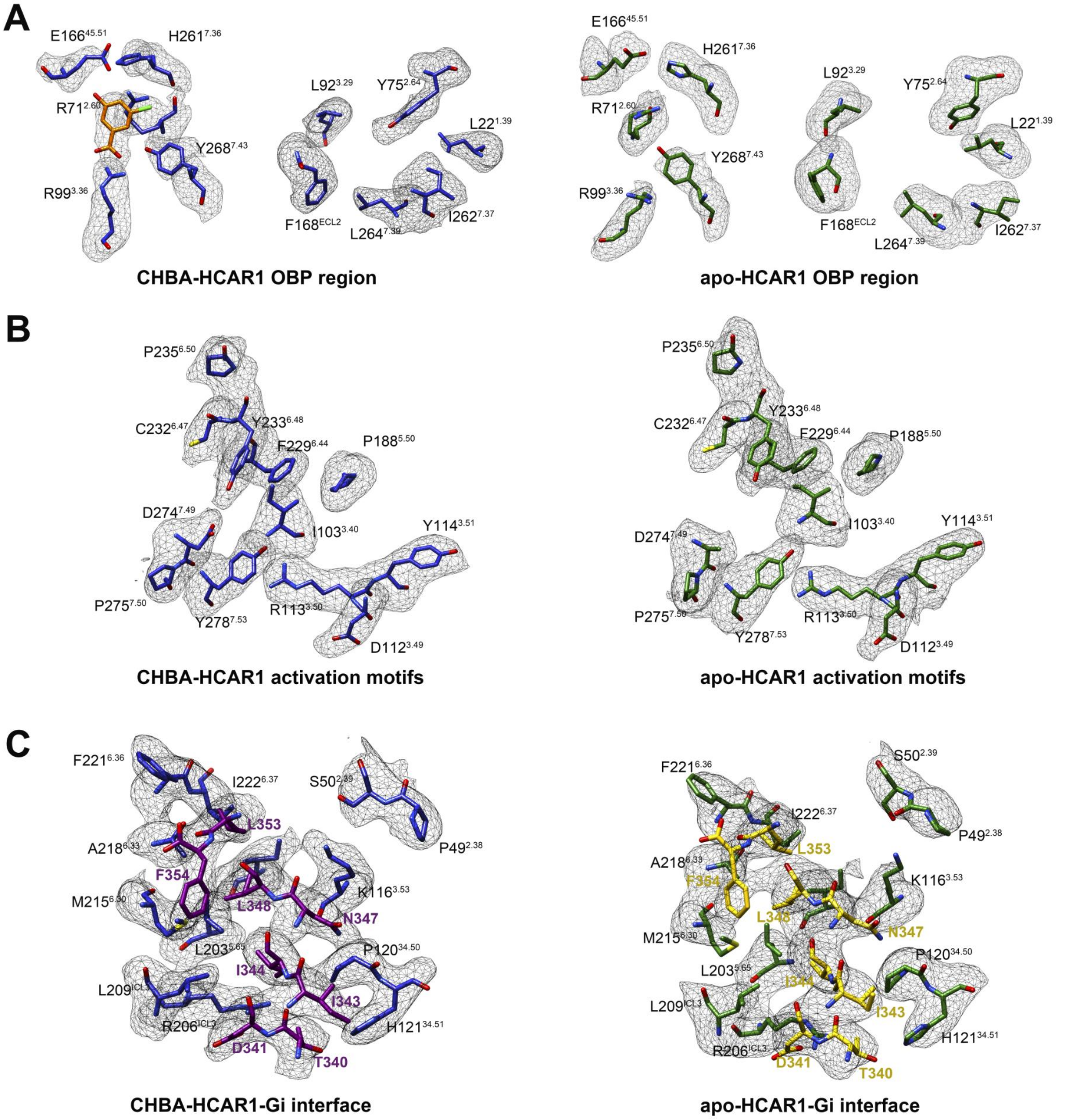

S6 Fig. Density maps of critical residues in the apo and CHBA-bound HCAR1. (A) Density maps of hydrophilic and hydrophobic residues in the OBP region. (B) Density maps of key activation

motifs. (C) Density maps of key residues in the HCAR1-Gi interface. Royal blue-dark magenta, CHBA-HCAR1-Gi1; forest green-beige, apo-HCAR1-Gi1; orange, CHBA.
