# Supplementary material for: Structures of G-protein coupled receptor HCAR1 in complex with Gi1 protein reveal the mechanistic basis for ligand recognition and agonist selectivity
Source: PLoS Biol. 2025 Apr 15;23(4):e3003126. doi: 10.1371/journal.pbio.3003126 (PMC12040280; doi:10.1371/journal.pbio.3003126)
Supplement: Attachment — Submitted filename: Response letter-PLOS Biology.doc [file pbio.3003126.s024.doc]

Dear Editors and Reviewers,

We would like to submit our revision for manuscript PBIOLOGY-D-24-02983R3 entitled “**Structures of G-protein coupled receptor HCAR1 in complex with Gi1 protein reveal the mechanistic basis for ligand recognition and agonist selectivity**”. We really appreciate the time both you and the reviewers have taken to detail your helpful comments and suggestions. Below you will find a list of changes for the point-by-point responses to the reviewers’ comments and a list of answers to the questions on the checklist.

In addition, we have carefully rechecked the entire manuscript, and any modifications have been highlighted in yellow in the revised version. We hope that the quality of our manuscript would meet the publication standard of ***PLOS Biology.***

Sincerely,

Yang Du, PhD, Assistant Professor/PI

School of Medicine

Kobilka Institute of Innovative Drug Discovery

The Chinese University of Hong Kong,Shenzhen 518172, China

**List of Changes**

**(**Manuscript ID: PBIOLOGY-D-24-02983R3, Pan et al.**)**

**Response to Editors**

**Question 1**. We routinely suggest changes to titles to ensure maximum accessibility for a broad, non-specialist readership. In this case, we would suggest a minor edit to the title, as follows. Please ensure you change both the manuscript file and the online submission system, as they need to match for final acceptance:“Structures of G-protein coupled receptor HCAR1 in complex with Gi1 protein reveal the mechanistic basis for ligand recognition and agonist selectivity”
**Answer 1**. Thank you very much for your valuable suggestion. According to your requirement, the title of the manuscript has been revised to **“Structures of G-protein coupled receptor HCAR1 in complex with Gi1 protein reveal the mechanistic basis for ligand recognition and agonist selectivity”.**

**Question 2**. Thank you for providing the structural data in the PDB and EMDB databases. However, we note that the data is currently on hold for release. We ask that you please make the structures publicly available at this stage before publication.

**Answer 2**. Thank you very much for your valuable suggestion. According to your requirement, we have contacted the PDB databases and made the structural data publicly available soon.

**Question 3**. Please also ensure that each of the relevant figure legends in your manuscript include information on *WHERE THE UNDERLYING DATA CAN BE FOUND*, and ensure your supplemental data file/s has a legend.

**Answer 3** Thank you very much for your valuable suggestion. All figure legends have been included in our manuscript.

Fig 1-Fig 7: Please see line 715-789

S1 Fig-S17 Fig, S1 Table, S1 Raw Images, S1 Data: Please see line 553-660

**Question 4**. Per journal policy, if you have generated any custom code during the course of this investigation, please make it available without restrictions. Please ensure that the code is sufficiently well documented and reusable, and that your Data Statement in the Editorial Manager submission system accurately describes where your code can be found.

**Answer 4**. No custom code is generated during the course.

**Question 5**. As you address these items, please take this last chance to review your reference list to ensure that it is complete and correct. If you have cited papers that have been retracted, please include the rationale for doing so in the manuscript text, or remove these references and replace them with relevant current references. Any changes to the reference list should be mentioned in the cover letter that accompanies your revised manuscript.

**Answer 5**. Thank you very much for your valuable suggestion. We do not cite the retracted papers. All the reference formats have been rechecked and revised to ensure that they are complete and correct. (Please see line 792-1016)

**Response to Reviewer 3**
**Question 1**. The authors have addressed all concerns in a satisfactory manner. The only minor point that I could point to still is the quality of the new Figure 7 - I suspect the authors would be able to improve the figure quite a bit still just by making sure all of the letters / numbers are within the shapes, and the shapes are well aligned. But I would leave the necessity of such changes to the authors discretion. I think it is a great manuscript that in principle should acceptable for publication at this stage.

**Answer 1**. Thank you very much for your kind and professional comments and suggestions. According to your requirement, all of the letters / numbers have been within the shapes, and the shapes have been well aligned.

Before modification

After modification
